# AI-powered the toughest biohydrogels

**DOI:** 10.1016/j.bioactmat.2026.07.002

**Published:** 2026-07-06

**Authors:** Jiameng Yang, Tao Fu, Ke Yao, Weicheng Kong, Bokun Li, Wanqing Xu, Zhou Zhu, Ximin Yuan, Yong He

**Affiliations:** a State Key Laboratory of Fluid Power and Mechatronic Systems & Liangzhu Laboratory, School of Mechanical Engineering, Zhejiang University, Hangzhou, 310027, China; b The Second Affiliated Hospital of Zhejiang University, Zhejiang University, Hangzhou, 310027, China; c Zhejiang Key Laboratory of Additive Manufacturing Technology and Equipment, College of Mechanical Engineering, Zhejiang University, Hangzhou, 310027, China; d Dr. Li Dak Sum & Yip Yio Chin Center for Stem Cell and Regenerative Medicine, Zhejiang University, Hangzhou, 310027, China; e Department of Oral and Maxillofacial Surgery, The Second Affiliated Hospital of Zhejiang University School of Medicine, School of Stomatology and Key Laboratory of Oral Biomedical Research of Zhejiang Province, Hangzhou, Zhejiang, 310000, China; f State Key Laboratory of Oral Diseases, National Clinical Research Center for Oral Diseases, West China Hospital of Stomatology, Sichuan University, Chengdu, 610041, China

**Keywords:** Biomanufacturing, Tough hydrogel, Salting-out effect, 3D printing, Biological hydrogel

## Abstract

Hydrogel soft materials hold immense promise for applications ranging from bionic soft robots to flexible human-machine interfaces, but realizing this potential critically depends on excellent mechanical properties. While substantial progress has been made in toughening hydrogels, concurrently achieving a significant enhancement in strength remains a formidable challenge, thereby limiting their functional use. This work introduced a CPTR (centrifugation - progressive training - restorative soaking) strategy, which prestructured the material through centrifugation, continuously evolved and optimized the structure through progressive training, and further refined and locked the structure through soaking. The mechanical properties were improved through the three processes synergistically. By inputting the process parameters and corresponding mechanical test results, the AI model analyzed feature importance, ranked optimal performance combinations, and recommended new schemes. Through such iterative cycles, a CPTR hydrogel with an outstanding tensile strength of 134.31 MPa and toughness of 10.25 MJ/m^3^ was achieved, currently the highest strength among biohydrogels. Its excellent mechanical performance and processability allowed the hydrogel to be constructed into fibers and network structures, opening new avenues for developing next-generation soft actuators, robust controllable release systems, and other advanced functional materials.

## Highlights

• We prepared the highest tensile strength biohydrogels (134.31 MPa) through a synergistic program of centrifugation-progressive training-restorative soaking.
• AI integration provided feedback on preparation schemes, understood the relationship between process and performance, analyzed feature importance, predicted optimal performance combinations, and recommended next step schemes for optimization and iteration to achieve the most precise parameter combinations.
• The hydrogel fibers and network structures built based on this provided broad application prospects in industries such as manufacturing and regenerative medicine.

## Graphical abstract

**Figure:**
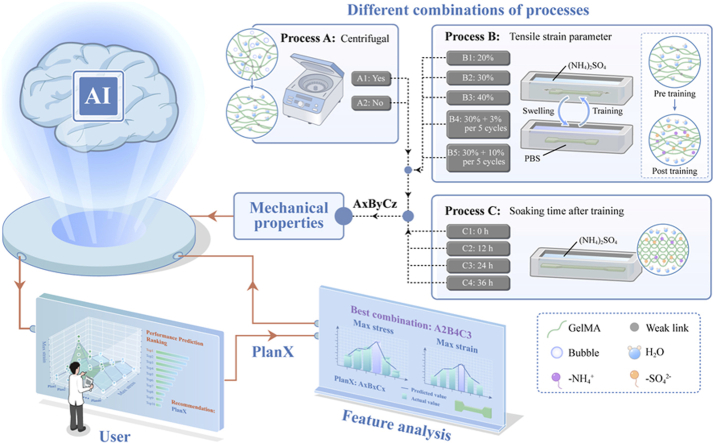

## 1 Introduction

Conventional hydrogels, such as alginate and gelatin, typically exhibit weak mechanical properties (<10 kPa) due to inhomogeneous crosslinking, driving sustained efforts to enhance their strength and toughness [[1], [2], [3]]. Since the 2000s, tough hydrogels have advanced rapidly [[4], [5], [6]], and their excellent mechanical properties have enabled widespread applications in biomedicine, tissue engineering [[7], [8], [9], [10], [11], [12], [13]], and flexible electronics [14,15]. Existing strategies for preparing tough hydrogels can be broadly grouped into three categories: (1) Dynamic sacrificial interactions: Introducing reversible sacrificial bonds (e.g., ionic bonds, hydrogen bonds, host–guest interactions) to dissipate energy at crack tips, thereby enhancing fracture toughness [[16], [17], [18], [19], [20], [21]]. (2) Intrinsic network optimization: Constructing uniform topological networks [22,23] or incorporating molecular pulleys [24,25] to reduce stress concentration and improve inherent strength and stretchability. (3) Multi-scale heterogeneous structures: Mimicking nature's hierarchical ordered structures, techniques such as mechanical training [[26], [27], [28]], composites [29,30], freeze casting [[31], [32], [33]], and mesoscale modifications [34] are employed to build fiber, layered, or honeycomb architectures from the nano-to the macroscale, effectively deflecting cracks to enhance overall mechanical properties. However, in addition to the shortcomings of complex processes, high costs, and long time consumption [[35], [36], [37], [38], [39], [40]], as well as the relatively low upper limit of final mechanical properties, poor iterability and small improvement in mechanical properties are also key issues [[41], [42], [43], [44], [45]].

Inspired by fitness, we developed a synergistic centrifugal-progressive training-restorative soaking (CPTR) strategy. The GelMA precursor solution was centrifuged (warm-up), condensed, crosslinked, and then subjected to cyclic axial loading in saline with stepwise strain increases (progressive anaerobic training), alternating with PBS immersion, followed by a prolonged saline soak (excess recovery); the resulting hydrogels are termed CPTR hydrogels. The process parameters and mechanical results were analyzed by an AI model, which provided feature importance rankings, optimal performance combinations, and design recommendations. Experiments were iteratively conducted by feeding the latest results back into the AI to refine subsequent trials. This cycle was repeated until the optimal process combination was achieved: centrifugation - starting with 30 % strain and increasing by 3 % strain every 5 cycles - soaking in saline for 24 h. The tensile strength reached up to 134.31 MPa, the toughness and the toughness normalized by polymer density were 10.25 MJ/m3 and 10.17 J/g, respectively (Fig. S19–20, Supporting In**formation)**. Furthermore, we fabricated hydrogel fibers and networks that exhibited excellent mechanical performance in practical tests, highlighting their significant potential for regenerative medicine and tissue engineering.

## 2 Results and discussion

### 2.1 Design of AI-feedback scheme optimization

With unique technology, tough hydrogels were produced efficiently and stably. In short, the hydrogel solution, dissolved in a constant temperature water bath at 55 °C, was centrifuged, then placed in a 4 °C incubator for condensation. After condensation, it was cured under a UV lamp, demolded, progressively stretched in ammonium sulfate solution, briefly soaked in PBS, and this cycle was repeated 20 times, after which it was soaked in ammonium sulfate solution for a long period.

**Fig. 1:**
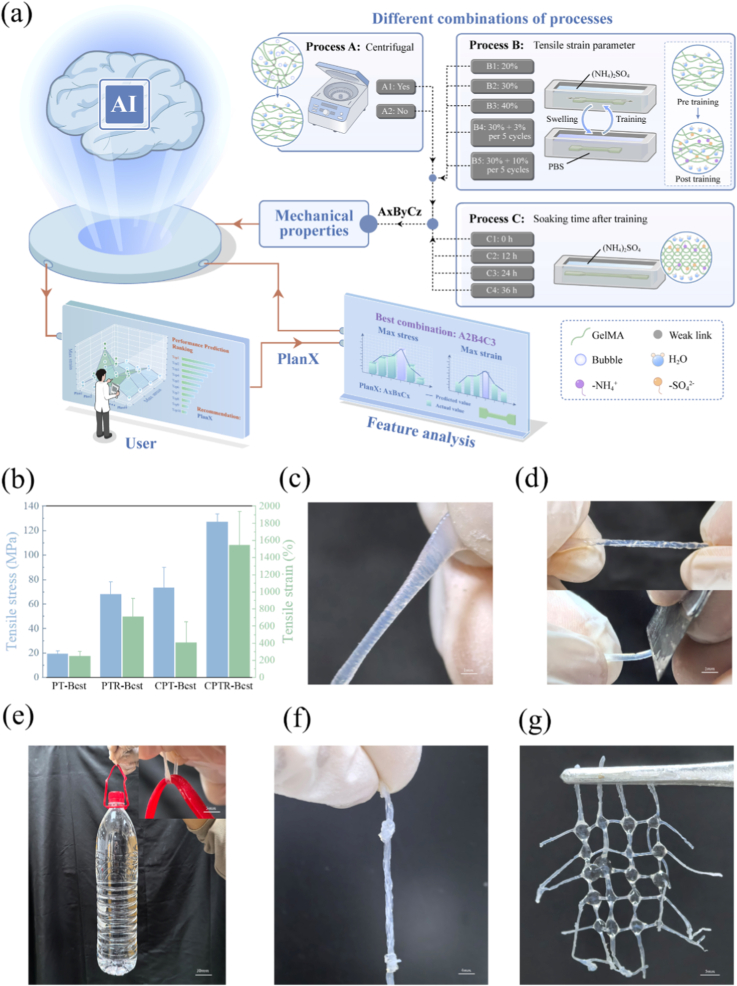
Schematic and results of AI-powered preparation of tough hydrogels. **a.** CPTR hydrogel obtained after repeated optimization by AI. **b.** Comparison of mechanical properties under the best parameters among four process combinations. **c.** Completed CPTR hydrogel. **d.** CPTR hydrogel enduring twisting and blade cutting. **e.** CPTR hydrogel used for weightlifting. **f.** Multistrand fibers of CPTR hydrogel. **g.** CPTR hydrogel network. (“PT” refers to biohydrogels prepared by progressive training approach. “PTR” refers to biohydrogels prepared by progressive training-restorative soaking approach. “CPT” refers to biohydrogels prepared by centrifugal-progressive training approach. “CPTR” refers to biohydrogels prepared by centrifugal-progressive training-restorative soaking approach).

Leveraging machine learning, we established a closed-loop optimization system: process parameters and corresponding mechanical results were iteratively fed into models, where random forest (RF) performed feature importance analysis, Gaussian process regression (GPR) ranked optimal process combinations, and Bayesian optimization (BO) recommended the next experimental step. The updated results were continuously looped back, enabling intelligent, dynamically adjusted preparation of tough hydrogels (Fig. 1a). Therefore, this AI-feedback driven process optimization provided technical support for improving the mechanical properties of hydrogels using CPTR.

### 2.2 Preparation and characterization of tough biohydrogel

Inspired by anaerobic muscle training—warm-up, progressive loading of a target muscle group, and supercompensation recovery—we developed a facile method to obtain tough biohydrogels. The GelMA precursor solution was centrifuged to realign molecular chains, restructure weak-interaction zones, and remove bubbles, then cast, condensed, photopolymerized, demolded, and immersed in saline for progressive axial cyclic stretching as mechanical training. According to the Hofmeister effect [35,36], under the influence of SO_4_^2−^, the preconcentrated hydrogel chains strongly self assembled and phase separated from the original homogeneous phase. Progressive increases in stretching strain continuously enhanced network orientation and promoted exposure of reactive sites. Cyclic stretching in saline alternating with PBS immersion strengthened interchain binding, and subsequent prolonged saline soaking provided a deep, less disruptive opportunity for crosslinking. A comparison of the best sets from the four process combinations—PT (progressive training), PTR (progressive training + restorative soaking), CPT (centrifugal + progressive training), and CPTR (centrifugal + progressive training + restorative soaking)—is shown (Fig. 1b), our strategy could increase the strength of ordinary hydrogels by 2920 times, achieving a maximum tensile stress of 134.31 MPa. Meanwhile, we conducted a performance comparison based on the more typical tough hydrogels among different strategies [19,26,28,40,43,45,46] (Fig. S9 and S18, Supporting Information**)**. Compared with the initial hydrogel, the CPTR hydrogel clearly exhibited orderly internal training marks (Fig. 1c). CPTR did not fracture under multiple twists or heavy blade pressing (Fig. 1d) and could lift a 1.5 L bottle of mineral water weighing 1.58 Kg with a cross-sectional area of only 0.306 mm^2^(Fig. 1e). In addition, hydrogel fibers (Fig. 1f) and hydrogel networks (Fig. 1g) could be produced under the CPTR strategy. Additionally, we conducted fatigue tests on CPTR hydrogel and toughness tests on hydrogels treated with four different process combinations. The results indicate that CPTR has excellent fatigue resistance and toughness (Fig. S10–S11, Supporting Information). And by comparing the tensile stress and Young's modulus of hydrogels in recent research progress on tough hydrogels and representative tough hydrogels in other strategies, we could see that the CPTR biohydrogels in this paper has higher tensile stress than other hydrogels, and its overall performance was better (Fig. S9, Supporting Information).

To elucidate the mechanisms underlying the property enhancement, we employed a multi-scale characterization strategy. At the microscale, SEM visualized the network pore structure. SAXS quantified the size, shape, and spatial arrangement of polymer aggregates; global ellipticity analysis of 2D patterns compared macroscopic network orientation across processes, and Porod analysis of 1D curves revealed the fractal morphology of the network's building blocks. Molecular-scale ordering and network uniformity were probed by WAXS and XRD. Complementary vibrational spectroscopy (FTIR and Raman) identified characteristic functional groups and provided evidence of secondary structures and intermolecular interactions. Finally, thermal properties were assessed by DSC and TG, whose thermal transitions, degradation behavior, and stability reflected the integrity of network crosslinking and ordered structures.

**Fig. 2:**
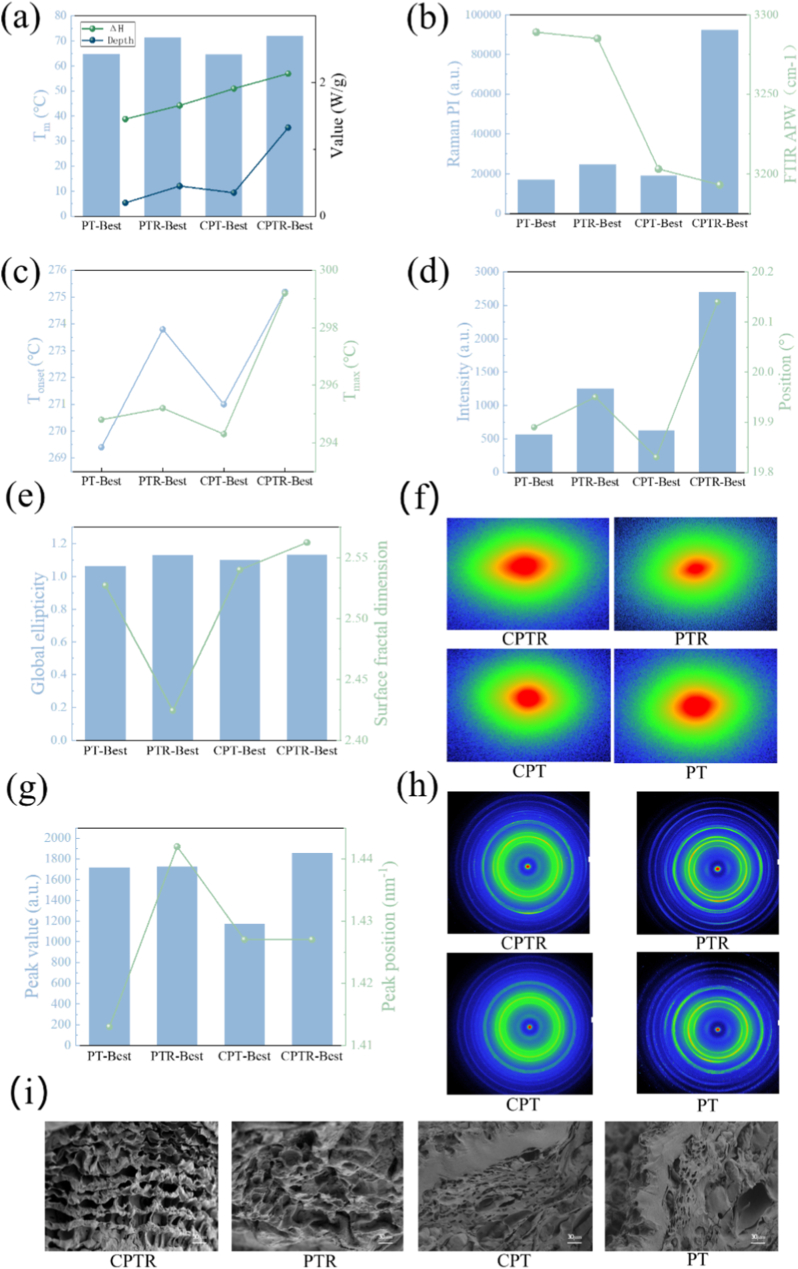
Characterization results of hydrogels properties under different process combinations. **a.** DSC characterization of hydrogels. **b.** Raman and FTIR characterization of hydrogels. **c.** TG characterization of hydrogels. **d.** XRD characterization of hydrogels. **e, f.** SAXS characterization of hydrogels **g, h.** WAXS characterization of hydrogels. **i.** SEM characterization of hydrogels.

The mechanical properties of CPTR and its mechanism was demonstrated and explained (Fig. 2). A sample with better mechanical performance had a more stable and ordered network structure, which showed higher phase transition temperature and greater phase transition enthalpy on the DSC curve. The melting temperature, melting enthalpy and peak depth of CPTR all exhibited better values (Fig. 2a, Fig. S1, Supporting Information). Raman characterization reflected changes in the protein secondary structure and the microenvironment of specific amino acid side chains. Centrifugation made the network more uniform, while progressive stretching training and prolonged static treatment with ammonium sulfate induced dehydration through salting out, causing the loose GelMA molecular chains to collapsed and formed dense nano aggregates stabilized by strong hydrogen bonds and hydrophobic interactions, resulting in higher peak intensity of the S-O bond vibration of the sulfate ions (Fig. 2b, Fig. S2, Supporting Information**)**. In FTIR infrared spectra, the vibration type of the amide A band was mainly N-H stretching vibration. When the N-H group participated in hydrogen bonding, its stretching vibration frequency decreased, and the peak became broader and stronger. Due to the dehydration effect of ammonium sulfate and polymer chain aggregation, the polymer chains were forced closer together, forming a large number of stronger interchain hydrogen bonds. Centrifugation also brought the advantage of homogenization, jointly promoting a redshift **(**Fig. S3, Supporting **Information)**. The denser and more stable the hydrogel network structure, the stronger the interactions between molecular chains, requiring a higher temperature to break these chemical bonds and decompose them. Therefore, hydrogels showed a higher initial decomposition temperature and a temperature for the maximum weight loss rate on the TGA curves (Fig. 2c, Fig. S5–S6, Supporting **Information)**. XRD characterization showed that hydrogels under the four process combinations had an amorphous halo peak around a diffraction angle of 20° with changes in position (Fig. 2d, Fig. S4, Supporting Information). The higher the peak intensity and the more the peak shifts to a higher angle, the tighter the molecular chain arrangement, indicating a higher network density and uniformity. In SAXS characterization, overall ellipticity described macroscopic orientation, and the Porod slope described microscopic morphology. The greater the ellipticity, the higher the degree of orientation, corresponding to better macroscopic mechanical properties. A higher Porod slope indicated a more complex internal fractal network, allowing internal stress to be more effectively dispersed over a wider area, reducing crack propagation and local damage (Fig. 2e, f, Fig. S17, Supporting Information). In WAXS characterization, the amorphous network peak of CPTR became stronger, indicating a smaller average distance between internal molecular chains and a higher network density (Fig. 2g and h). SEM characterization results showed that compared with PT, CPT, and PTR, the CPTR had more uniform network wall thickness and density, smaller pore size and more uniform distribution, with fewer cavities, microcracks, and other defect areas, reflecting higher crosslinking density and aggregation of internal molecular chains, fewer stress concentration points, and a more uniform structure (Fig. 2i, Fig. S17, Supporting Information).

### 2.3 Strengthening mechanism

**Fig. 3:**
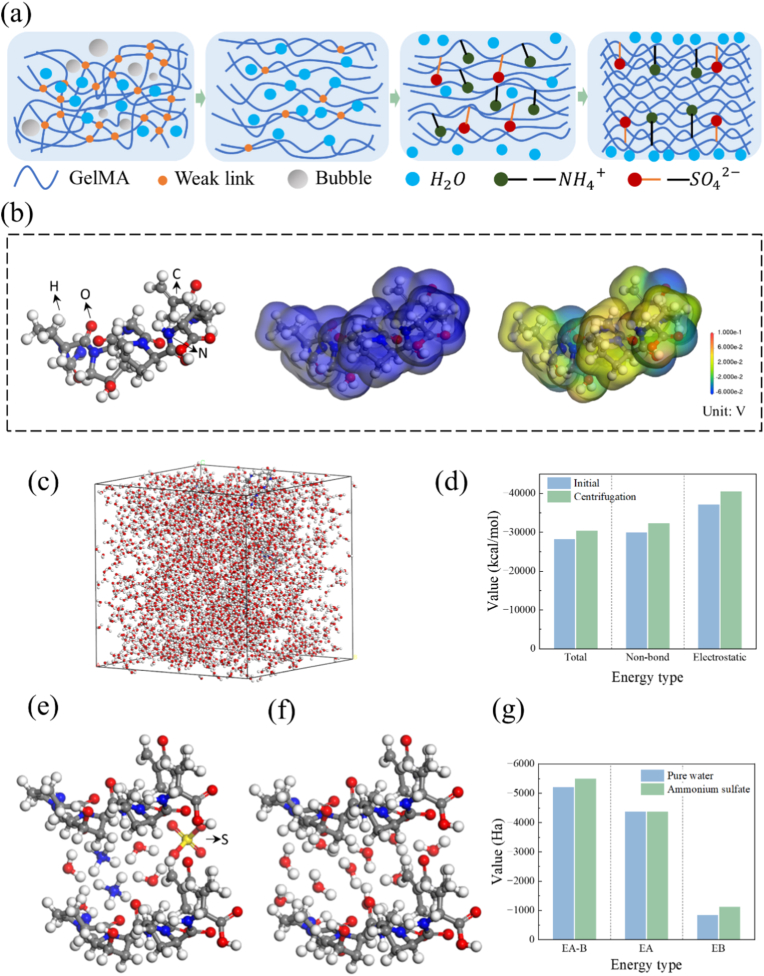
Reinforcement mechanism of CPTR biohydrogels. **a.** Structural changes inside the initial hydrogel after C (centrifugal), PT (progressive training), and R (restorative soaking) treatment. **b.** Molecular charge, electron density, and electrostatic potential of GelMA chains. **c.** Centrifugation process of hydrogels. **d.** Energy comparison of hydrogels before and after centrifugation. **e.** Hydrogels in an ammonium sulfate environment. **f.** Hydrogels in a pure water environment. **g.** Energy comparison of the hydrogels under the two environments.

The GelMA molecular chain was composed of gelatin and methacryloyl groups, with its basic structural unit being amino acid residues, which were long chain polymers formed by linking various amino acids through peptide bonds. Its amino acid sequence contained a large number of repeating Glycine-Proline-Hydroxyproline (Gly-Pro-Hyp) tripeptide units, and the core of the study was methacrylation modification, which mainly occurred on the amino group (ε-amino) at the end of the lysine side chain. Based on the above, a grafted MA hexapeptide (Gly-Pro-Hyp)-(Gly-Pro-Lys) containing both structural motifs and reactive sites could be constructed as a GelMA molecular chain(Fig. 3b).

GelMA chains, rich in polar groups (amino, hydroxyl, carboxyl), promoted numerous weak intra- and inter-chain connections. As semiflexible chains in static solution, they adopted randomly coiled conformations with entrapped bubbles, forming weak interaction zones through transient hydrogen bonds, hydrophobic interactions, and ionic bonds. This led to local concentration inhomogeneity and defect points upon subsequent crosslinking (Fig. 3a).

During centrifugation, centrifugal force overcame Brownian motion and conformational entropy, radially stretching and disentangling randomly coiled chains, while the induced shear flow further aligned them. This combined action disrupted weak interaction zones, densified and homogenized chain entanglements, and drove bubbles toward the central axis to dissipate, thereby reducing stress concentrators and structural defects in the subsequently cured network.

Geometric optimization using the Forcite module simulated centrifugation. Comparisons of total, non-bonded, and electrostatic energies revealed that centrifugation released energy, promoting more favorable hydrogen bonds and hydrophobic interactions. Molecular rearrangement strengthened attractive charge-center interactions, and the chains adopted positions more stable than the initial structure (Fig. 3c and d).

Progressive training forced the system to surmount energy barriers with each strain increment, driving it toward more stable configurations. Chain orientation increased along the stretching direction, yielding denser physical crosslinks. After each increment, stress relaxed and redistributed uniformly, preventing destructive stress concentrations. Subsequent cycles selectively disrupted weaker bonds formed earlier, promoting stronger ionic bonds, hydrogen bonds, and hydrophobic stacking, while larger strains exposed previously buried hydrophobic segments and polar groups, providing additional sites for salt-bridge and ionic bond formation with ammonium sulfate ions (SO_4_^2−^, NH_4_^+^) and further elevating physical crosslink density.

Prolonged soaking permitted full thermodynamic relaxation, allowing oriented chains to reorganize into more ordered and compact interaction sites. The uninterrupted salting-out effect drove SO_4_^2−^ and NH_4_^+^ to continuously compete for water molecules, inducing further chain dehydration, strengthening hydrophobic interactions and hydrogen bonds, and reducing inter-chain spacing while enhancing entanglement. Under the residual tension from mechanical training, these ions formed stable salt bridges and ionic bonds with chain functional groups, collectively building a denser, more stable physical crosslinking network (Fig. 3e–g).

### 2.4 Swelling characteristics test

**Fig. 4:**
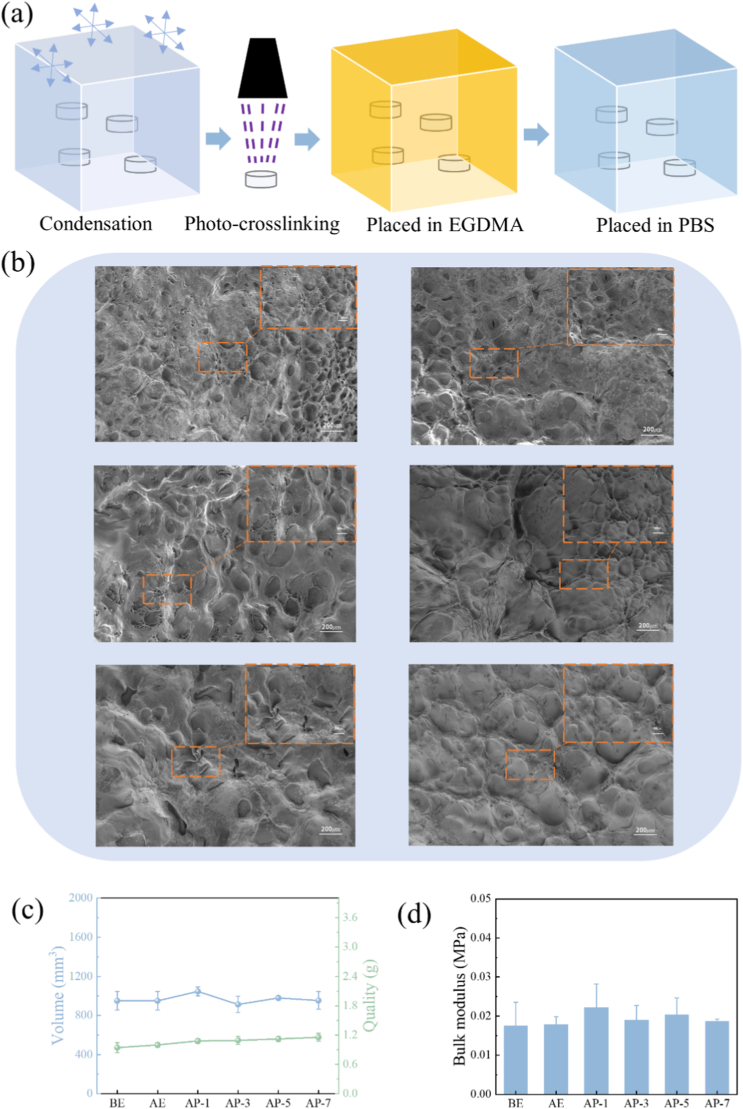
Swelling Invariance Test. **a.** Schematic diagram of the swelling experiment process. **b.** Comparison of the microscopic morphology before soaking in EGDMA, after soaking in EGDMA, and after soaking in PBS for 1, 3, 5, and 7 days. **c.** Changes of volume and mass. **d.** Changes of compression modulus. (“BE” refers to CPTR biohydrogels before soaking in EGDMA. “AE” refers to CPTR biohydrogels after soaking in EGDMA. “AP-1” refers to CPTR biohydrogels after being soaked in PBS for 1 days. “AP-3” refers to CPTR biohydrogels after being soaked in PBS for 3 days. “AP-5” refers to CPTR biohydrogels after being soaked in PBS for 5 days. “AP-7” refers to CPTR biohydrogels after being soaked in PBS for 7 days).

Cylindrical molds were made using PLA material, with a diameter of 22 mm, a height of 4 mm, and a thickness of 1 mm. Based on this, hydrogel samples used in this experiment were prepared. After condensation and photopolymerization, the molds were demolded and soaked in ethylene glycol dimethacrylate (EGDMA) for 5 min, then soaked in PBS(Fig. 4a). The volume, mass, and compressive modulus were measured sequentially before and after EGDMA soaking, and after soaking in PBS for 1 day, 3 days, 5 days, and 7 days (Fig. 4c and d). The microstructural changes were observed via SEM (Fig. 4b). The results showed that throughout the entire process, the changes in sample volume, mass, compressive modulus, and microstructure were minimal, indicating that the samples exhibit swelling invariance. In addition, based on the tests of water content for the four groups of CPTR, CPT, PTR, and PT, as well as measurements of density and volume changes before and after soaking in EGDMA, and after being immersed in PBS solution for 1 day and 3 days, we found that the best group (CPTR) has the lowest water content, the highest density, and the smallest volume change (Fig. S13–S15, Supporting Information). Therefore, the different processing steps were related to the increase in polymer density, and the groups with better mechanical properties also have better swelling invariance. However, by comparing the mechanical properties of biohydrogels under different process combinations normalized by polymer density (Fig. S20–S26, Supporting **Information)**, it can be seen that the enhancement of the mechanical properties of biohydrogels does not entirely come from an increase in polymer density, but mainly from processing-induced network reconstruction, chain segment orientation, the reinforcement of physical crosslinking, improved energy dissipation efficiency and so on.

Moreover, we conducted CCK-8 assays and cell viability and death staining experiments on CPTR biohydrogels before and after EGDMA immersion. After 1 and 3 days of culture, there was no significant difference in cell viability between the experimental group and the control group. The cell viability and death staining results also showed few red-stained dead cells, indicating that the hydrogel has good cytocompatibility (Fig. S16, Supporting Information).

### 2.5 AI-assisted experiment

**Fig. 5:**
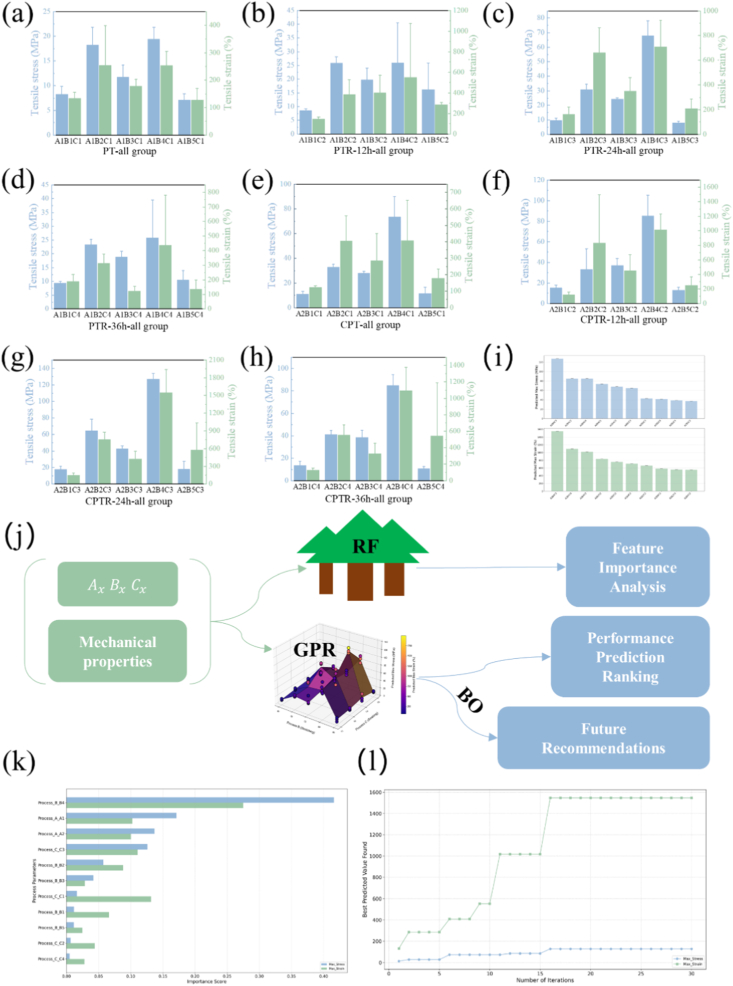
Comparison of Mechanical Properties Under Different Process Combinations and AI Analysis Results. **a.** Comparison of performance for all PT combinations. **b.** Comparison of performance for all PTR-12h combinations. **c.** Comparison of performance for all PTR-24h combinations. **d**. Comparison of performance for all PTR-36h combinations. **e.** Comparison of performance for all CPT combinations. **f.** Comparison of performance for all CPTR-12h combinations. **g.** Comparison of performance for all CPTR-24h combinations. **h.** Comparison of performance for all CPTR-36h combinations. **i.** Ranking of the best performance combinations of GPR. **j.** AI model optimization process. **k.** RF feature importance ranking. **l.** BO recommended plan. (A1: no centrifugation. A2: centrifugation. B1: 20 % strain stretching. B2: 30 % strain stretching. B3: 40 % strain stretching. B4: initial 30 % strain with an increase of 3 % strain every 5 cycles. B5: initial 30 % strain with an increase of 10 % strain every 5 cycles. C1: no soaking after stretching. C2: soaking for 12 h after stretching. C3: soaking for 24 h after stretching. C4: soaking for 36 h after stretching).

We encoded the centrifugation process as A, no centrifugation as A1, and centrifugation as A2. Encoded the stretching training process as B, 20 % strain stretching as B1, 30 % strain stretching as B2, 40 % strain stretching as B3, initial 30 % strain with an increase of 3 % strain every 5 cycles as B4, and initial 30 % strain with an increase of 10 % strain every 5 cycles as B5. Encoded the soaking process as C, no soaking after stretching as C1, soaking for 12 h after stretching as C2, soaking for 24 h after stretching as C3, and soaking for 36 h after stretching as C4. There were a total of 40 process combinations, and We obtained the effects of different processing steps on intermolecular interaction forces through bidirectional verification of simulation and experiments, which in turn affected the final mechanical properties. (Fig. 5a–h and Figure S7-S8, S12, Supporting Information**)**.

Traditional optimization treated parameter combinations as discrete data points, hindering factor contribution quantification and effective experimental guidance. To overcome this, we proposed a machine-learning-driven strategy: random forests identified key process factors, Gaussian process regression modeled performance and uncertainty across the entire parameter space, and Bayesian optimization intelligently searched for optimal processes. This analyze–predict–decide closed-loop approach transformed data use from simple performance ranking to forward-looking guidance (Fig. 5j).

In the random forest (RF) model, the forest was constructed based on bootstrap aggregation sampling and feature randomness, followed by training of decision trees to collectively make decisions for subsequent process inputs, taking the average of all tree predictions as the final result and obtaining the feature importance ranking. This approach reduced model variance and enhances generalization capability (Fig. 5i). In the Gaussian process regression (GPR) model, the model was configured using a combination of RBF Kernel and Constant Kernel. the former established a smooth, nonlinear relationship to learn the overall trend of how process combinations affect performance, while the latter accounted for the noise in the data. Since each process combination had three experimental data sets, this kernel could learn and explain such random fluctuations, enabling efficient and comprehensive predictions (Fig. 5k). In the Bayesian optimization (BO) model, the trained GPR model was used as a surrogate model, and the acquisition function (Expected Improvement, EI) was applied to iteratively approach the optimal solution (Fig. 5l). The regression coefficients were calculated as 0.833 for RF and 0.814 for GPR, indicating excellent analytical accuracy.

All original experimental data were used for model training. This dataset contains 120 experimental records, covering all 40 possible combinations of three categorical process parameters, with each combination independently repeated three times. Due to the relatively small sample size, and since the main purpose of this study is to explore the existing experimental space and recommend potentially new process combinations, we used the full dataset for model training. We used the leave-one-out cross-validation method. The random forest predicted the maximum stress with R^2^ = 0.8833. Gaussian process regression predicted the maximum stress with R^2^ = 0.8774. The stress model demonstrates good predictive ability, confirming that the mapping relationship between process parameters and stress is effectively captured. In the RF model, We set the number of decision trees to 100 and the random seed to 42, with the other parameters using sklearn defaults. In the GPR model, We used the Constant Kernel and RBF kernel as the kernel function. The initial value of the Constant Kernel was set to 1.0, with its automatic optimization range limited between 0.001 and 1000. The initial value of the RBF kernel was set to 1.0, with an automatic optimization range from 0.01 to 100. And we set the number of optimizer restarts to 10 to avoid local optima, with random seed 42. In the BO model, the expected improvement acquisition function was used, with a total of 30 function evaluations, and random seed 42. The optimization process was carried out on the surface of the trained GPR surrogate model to recommend the best candidate combination.

### 2.6 Preparation of biohydrogel fibers and networks

**Fig. 6:**
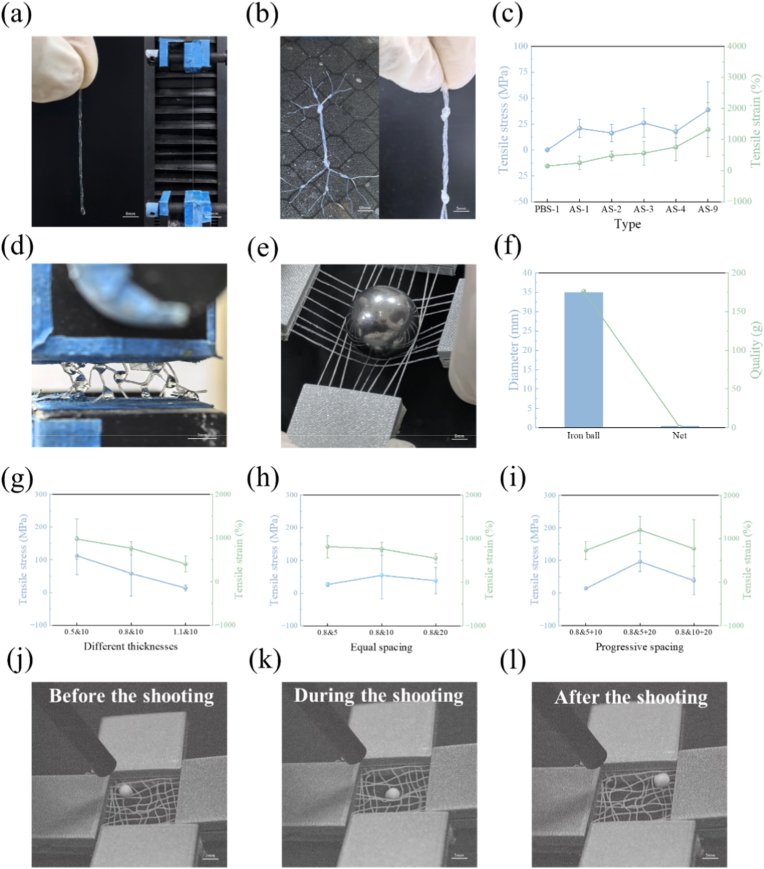
Mechanical performance testing and applications of CPTR hydrogel fibers and networks. **a.** Schematic of a single hydrogel fiber. **b.** Preparation of a nine strand hydrogel fiber. **c.** Comparison of mechanical properties of hydrogel fibers with different strands. **d.** Mechanical performance testing of hydrogel networks. **e.** Hydrogel network used to support an iron ball. **f.** Comparison of the mass and diameter of the hydrogel network and the supported iron ball. **g.** Differences in mechanical properties of hydrogel networks composed of fibers with different diameters. **h.** Differences in mechanical properties of hydrogel networks with different uniform spacing. **i.** Differences in mechanical properties of hydrogel networks with progressively spaced pores. **j, k, l.** Process images of bullets striking hydrogel networks. (Single, double, triple, quadruple, and nine strand fibers are respectively referred to as AS-1, AS-2, AS-3, AS-4, AS-9, and single fibers soaked only in PBS are referred to as PBS-1).

Using a disposable sterile syringe, the hydrophobic modification solution(fluoro modified acrylic acid) was injected into a transparent silicone tube. After letting it sit for a period of time, the centrifuged hydrogel precursor solution was injected into the transparent silicone tube. The hydrogel precursor solution inside the silicone tube was condensed and photochemically crosslinked. A metal adjustable continuous dispenser was used to draw in deionized water, then the hydrogels were extruded into an ammonium sulfate solution through the water pressure and for gradual training and settling, yielding tough single hydrogel fibers. Based on this, double, triple, quadruple, and nine strand fibers were produced (referred to as AS-2, AS-3, AS-4, AS-9, respectively) (Fig. 6a–c).

Using a single variable control method, the diameter of a single fiber at 0.8 mm was kept constant in the first group, with the network pore spacing set at equal intervals of 5 mm, 10 mm, and 20 mm. the second group kept the single fiber diameter at 0.8 mm but sets the network pore spacing at unequal intervals. the third group kept the hydrogel network pore spacing at a constant 10 mm with equal intervals, while the single fiber diameters were set at 0.5 mm, 0.8 mm, and 1.1 mm. The mechanical performance of the three groups of hydrogel networks were compared (Fig. 6d–g-i).

A solid iron ball with a mass of 176.23 g and a diameter of 35 mm was placed on the hydrogel network, and the network was able to fully support it (Fig. 6e and Movie S1, Supporting I**nformation)**. The mass of the iron ball was 367.15 times that of the network, and the diameter of the iron ball was 79.55 times that of the network (Fig. 6f). A plastic bullet traveling at 15.2 m/s with an average impact force of 2.964 N was shot at the network, and the network remained completely undamaged (Fig. 6f). The experimental settings of the high-speed camera were: resolution 640×480. Sampling rate 3000 pps. exposure time 99.525 ms. exposure index 400,000. The figures showed the process of the bullet striking the hydrogel network as captured by the high-speed camera (Fig. 6j–l **and Movie S2,** Supporting **Information**).

## 3 Conclusion

In summary, by combining processes and conducting experiments based on AI analysis and prediction results, we ultimately prepared a gelatin-based tough biohydrogel using the CPTR strategy, with a tensile strength of 134.31 MPa, making it the strongest ever in terms of tensile strength. In addition, we used this strategy to create biohydrogel fibers and biohydrogel networks with excellent mechanical properties. This expands the potential applications of tough hydrogels in fields such as tissue engineering and regenerative medicine.

## 4 Methods

### 4.1 Materials

Phosphate-buffered saline (PBS) was purchased from Jiangsu Kaiji Biotechnology Co., Ltd. Lithium phenyl-2,4,6-trimethylbenzoylphosphinate (LAP) was purchased from Huaxia Siyin Biotechnology Co., Ltd. GelMA (EFL-GelMA-30) was purchased from Suzhou Yongqinquan Intelligent Equipment Co., Ltd., China. Gelatin (G108395 gelatin, for microbiology, gel strength ≈ 250 g Bloom) was purchased from Aladdin. Ammonium sulfate ((NH_4_)_2_SO_4_, AR, 99 %) was purchased from Macklin. Methacrylic anhydride (MA, ≥94 %) was obtained from Hyclone, Hong Kong. Transparent silicone tubing was purchased from Huai'an Bofan Rubber & Plastic Business Department. Hydrophobic modification solution was purchased from Dongguan Zhongke Lingchuang Technology Co., Ltd. Acrylic rectangular uncovered trays were purchased from Shenzhen Langzhi Technology Co., Ltd. Solid iron balls were purchased from Yiwu Hongrui Hardware Co., Ltd. Deionized water was prepared using a laboratory water purification system.

### 4.2 Fabrication of tough Hydrogel(Fabrication of hydrogel solution)

The hydrogel used in this experiment was prepared based on GelMA30: 2 g of GelMA and 0.08 g of LAP were dissolved in 40 mL of PBS and poured into a centrifuge tube. The mixture was stirred and heated at 55 °C until completely dissolved, then balanced and centrifuged at 2000 rpm for 3 min. After centrifugation, the hydrogel solution was taken with a dropper and then poured into silicone molds used to make training samples. The molds were manufactured using an S140 printer (BMF), and the successfully printed molds were treated ultrasonically with ethanol and hydrofluoric acid. Next, PDMS (monomer and curing agent mixed at a 10:1 ratio and treated with a vacuum defoamer) was slowly poured into the cleaned molds and placed in an 80 °C oven to stand for 2 h. Then, an appropriate amount of prepared hydrogel solution was dripped into the PDMS. The mold was then placed in a 2-8 °C refrigerator and left for 30 min. Finally, the hydrogel was crosslinked and cured under a UV curing lamp and the cured hydrogel was removed. The hydrogel samples were wrapped at both ends with non-woven fabric to reduce damage from the clamps, placed in the clamps of the hydrogel training machine, and two rectangular liquid tanks were prepared, one filled with 50 % ammonium sulfate solution and the other with PBS solution. The clamps were first placed in the tank containing ammonium sulfate solution for stretching training, with an initial stretch strain of 30 % and 20 stretching cycles. After stretching, they were placed in PBS solution for 60 s, then returned to the ammonium sulfate solution for stretching training again. Every 5 cycles, the maximum stretch strain was increased by 3 %. After repeating this for 20 cycles, the samples were removed from the clamps and placed in ammonium sulfate solution for 24 h, then rinsed in PBS solution. Finally, the samples were placed on a tensile testing machine to test their mechanical properties.

### 4.3 Mechanical testing

In the tensile and toughness tests, we used a universal testing machine and the same tensile parameters, mechanical performance tests were conducted on hydrogels that were only centrifuged, hydrogels subjected to centrifugation and progressive tensile training, hydrogels subjected to progressive tensile training and long-term static treatment, and hydrogels subjected to centrifugation, progressive tensile training, and long-term static treatment. The reference standard code is GB/T 13022-1991, the type is set to tension, the tensile speed is set to 5 mm/min, and the control mode is set to displacement-controlled. In the fatigue test, the inlet force was set to 0.001 N, the number of cycles was 50, the speed was set to 30 mm/min, and the endpoint was set to 30 % of the initial length.

### 4.4 Structure simulation

Using Materials Studio software, the GelMA molecular chain model was constructed with the Visualizer module. the formulation was calculated based on the NetMass of the model, and then the centrifugal process was simulated using geometry optimization calculations in the Forcite module. the Energy task in the DMol3 module was used to simulate the comparison of GelMA chains in ammonium sulfate solution versus pure water.

### 4.5 Micromorphology characterization

After rinsing the hydrogel samples thoroughly, they were placed in a 4 °C environment for 1 h, then placed in a −20 °C environment for 2 h. Next, the samples were frozen and dried by using the SCIENTZ-18N/E (NINGBO SCIENTZ BIOTECHNOLOGY CO., LTD) lyophilizer. The lyophilizer was paired with an XZ-16 oil rotary vacuum pump (NINGBO SCIENTZ BIOTECHNOLOGY CO., LTD), with a frequency of 50/60 Hz, pumping speed of 240/290 L/min, ultimate pressure of 5 × 10^−1^ Pa, oil volume of 0.65-1.2 L, and motor power of 0.55 kW, 4 poles. The freeze-drying duration is 24 h. After freeze-drying, the samples were cut to expose the interior and sputter-coat them with gold to facilitate SEM imaging. The scanning electron microscope used was a Zeiss GeminiSEM 300. Experimental parameters were: voltage 3 kV, aperture 60 μm, probe SE2. The gold sputter coater used was Lebo, with parameters set to: vacuum 6 Pa, sputtering current 30 mA, sputtering time 25 s.

### 4.6 Vibrational spectroscopy characterization

FTIR: The lyophilized samples were placed in a Fourier Transform Infrared Spectrometer, model NICOLET iS50 FT-IR (manufactured by Thermo Scientific). Experimental parameters were set as follows: resolution 4 cm^−1^, 32 scans, scanning range from 4000 cm^−1^ to 400 cm^−1^.

Raman: The lyophilized samples were placed in a Renishaw multi-channel Raman spectrometer. Experimental parameters were set as follows: exposure time 1.000 s. Laser power set to 1000 %. Excitation wavelength set from 200 nm to 3800 nm.

### 4.7 Thermal analysis characterization

DSC: The freeze-dried samples were placed in the Mettler-Toledo Differential Scanning Calorimeter DSC 3. Experimental conditions were as follows: Under a nitrogen atmosphere, set the nitrogen flow rate to 50 mL/min. First, held at 10 °C for 3 min, then heated to 100 °C at a rate of 5 °C/min, held at 100 °C for 3 min, and then cooled to 10 °C at a rate of 5 °C/min.

TG: The freeze-dried samples were placed in the Mettler-Toledo Thermogravimetric Analyzer TGA/DSC 3. Experimental conditions were as follows: Under a nitrogen atmosphere, started at 30 °C and increased the temperature to 1000 °C at a rate of 10 °C/min.

### 4.8 Structural diffraction characterization

XRD: The freeze-dried samples were placed in a Bruker D8 Advance diffractometer (Cu-Kα radiation 1.540598 Å). The test conditions were set as follows: 2*θ* range of 5∼90°. Step size of 0.02°. Time per step of 0.1 s. The optical path conditions were set as: Primary Soller slit 4.1°/4.0°. Air scatter screen mode set to automatic. Receiving slit 0.5°. Voltage: 40 kV. Current: 40 mA. Detector: LYNXEYE XE-T.

SAXS and WAXS: The freeze-dried samples were placed in a small-angle X-ray scattering instrument, model XENOCS XEUSS 3.0∗ small angle X-ray scattering instrument. Experimental parameters were set as: Voltage: 70 kV. Current: 3.5 mA. Power: 250 W. Measurement time: 600 s. X-ray source: copper target. Detector model: Dectris EIGER2 Si 1M, using a point source. Detection wavelength: 0.154 nm. Pixel size: 75. Sample chamber maintained under vacuum, and no sample holder was used during measurement. For SAXS characterization, the S-D detector distance from the sample was 1500 mm. For WAXS characterization, the S-D detector distance from the sample was 42.5 mm.

### 4.9 Reliability of the AI-feedback scheme characterization

The mechanical properties of the hydrogel were verified using an electronic universal testing machine (UTM2102, Shenzhen Suntech Co., Ltd.) equipped with a 100N load cell.

### 4.10 Cell viability assay of CPTR biohydrogels

The CCK-8 method was used to evaluate the cytocompatibility of the hydrogel. The hydrogel was co-cultured with L929 cells, and after incubation for 1 day and 3 days, the culture medium was replaced with fresh medium containing 10 % CCK-8 reagent, followed by incubation in the dark for 2 h. Then, 150 μL of the solution from each well was transferred to a 96-well plate, and the absorbance was measured at 450 nm. All experiments were repeated three times.

The Calcein-AM/PI double staining kit (Beyotime, China) was used to further evaluate the cell compatibility of the hydrogel. Cells were co-cultured with the hydrogel. After 1 day and 3 days of incubation, the culture medium was aspirated, and the cells were gently washed with PBS. Mix 4 μL of Calcein-AM and 4 μL of PI with 1 ml of PBS to prepare the working staining solution. Then, 200 μL of the staining solution was added to each well and incubated at 37 °C in the dark for 30 min. Finally, the stained cells were visualized and imaged using an inverted fluorescence microscope.

### 4.11 Synthesis of hydrogel fibers

A 5 mL disposable sterile syringe was used to inject the hydrophobic modification solution (fluoroacrylic acid) into transparent silicone tubes with inner diameters of 0.5 mm, 0.8 mm, and 1.1 mm. A 5 mL disposable sterile syringe was used to inject the centrifuged hydrogel precursor solution into transparent silicone tubes with inner diameters of 0.5 mm, 0.8 mm, and 1.1 mm. The silicone tubes filled with hydrogel precursor solution were placed in a 4 °C constant temperature environment to condense for 30 min. The silicone tubes were removed and exposed to 405 nm light for crosslinking and curing (to photochemically cure the hydrogel inside the silicone tubes). A 50 mL adjustable metal continuous dispenser was used to draw in deionized water, then connected a syringe needle matching the inner diameter of the silicone tube to the dispenser nozzle and inserted the needle into the silicone tube, using water pressure to extrude the condensed and photo-cured hydrogel into ammonium sulfate solution, performing progressive training and prolonged soaking to obtain single hydrogel fibers. Based on this, two strand, three strand, four strand, and nine strand fibers could be made. The method for making three strand fibers was: arranged three fibers from left to right, named 1, 2, and 3. First, pressed fiber 1 from top to bottom onto fiber 2 and moved it to the middle on the right. Then, pressed fiber 3 from top to bottom onto fiber 2 and moved it to the middle on the left. Repeated this process to form a three strand fiber. The method for making four strand fibers was: arranged four fibers from left to right, named 1, 2, 3, and 4. First, pressed fiber 3 from top to bottom onto fiber 2 and moved it left. Then, lifted fiber 1 from bottom to top onto fiber 2 and moved it right. next, pressed fiber 4 from top to bottom onto fiber 3 and then moved it left under fiber 2. Then, pressed fiber 1 from top to bottom onto fiber 2 and moved it right. Next, lifted fiber 4 from bottom to top onto fiber 3 and moved it left over fiber 2. Repeated this process to form a four strand fiber. The method for making nine strand fibers was: First, braided three three strand fibers according to the three strand fiber method. Then, treated each three strand fiber as a single strand and braided them again according to the three strand fiber method to obtain a nine strand fiber.

### 4.12 Performance testing of hydrogel networks

Hydrogel fibers were arranged uniformly to form a hydrogel network, which supported a solid iron ball. The network was then struck by a bullet to demonstrate its mechanical properties. The bullet impact process was captured using the Phantom v2512 high speed camera from Vision Research, with the experimental settings as follows: resolution of 640×480. Sampling rate of 3000 pps. Exposure time of 99.525 ms. Sensitivity index of 400,000.

## Ethics approval and consent to participate

All experiments in this manuscript are limited to the fabrication, mechanical and multi-scale characterizations of GelMA-based biohydrogels, as well as in vitro cytocompatibility evaluation using commercially available L929 cell lines. No human subjects, patient samples, or live vertebrate animals were utilized in any experimental procedure. Accordingly, institutional ethics committee approval and informed consent are not necessary for this research.

## CRediT authorship contribution statement

**Jiameng Yang:** Conceptualization, Data curation, Formal analysis, Investigation, Methodology, Software, Validation, Writing – original draft, Writing – review & editing. **Tao Fu:** Investigation. **Ke Yao:** Investigation, Writing – original draft. **Weicheng Kong:** Investigation. **Bokun Li:** Conceptualization, Writing – original draft. **Wanqing Xu:** Investigation, Writing – original draft. **Zhou Zhu:** Supervision, Validation. **Ximin Yuan:** Conceptualization, Data curation, Formal analysis, Funding acquisition, Methodology, Resources, Supervision, Validation, Writing – review & editing. **Yong He:** Conceptualization, Data curation, Formal analysis, Funding acquisition, Investigation, Methodology, Project administration, Resources, Supervision, Visualization, Writing – review & editing.

## Declaration of competing interest

The authors declare no competing interests.
