# Supplementary material for: AI-powered the toughest biohydrogels
Source: Bioact Mater. 2026 Jul 6;66:339–51. doi: 10.1016/j.bioactmat.2026.07.002 (PMC13355382; doi:10.1016/j.bioactmat.2026.07.002)
Supplement: Multimedia component 1 [file mmc1.docx]

Supporting Information for

**AI-Powered the Toughest Biohydrogels**

**Supplementary Figures**

**
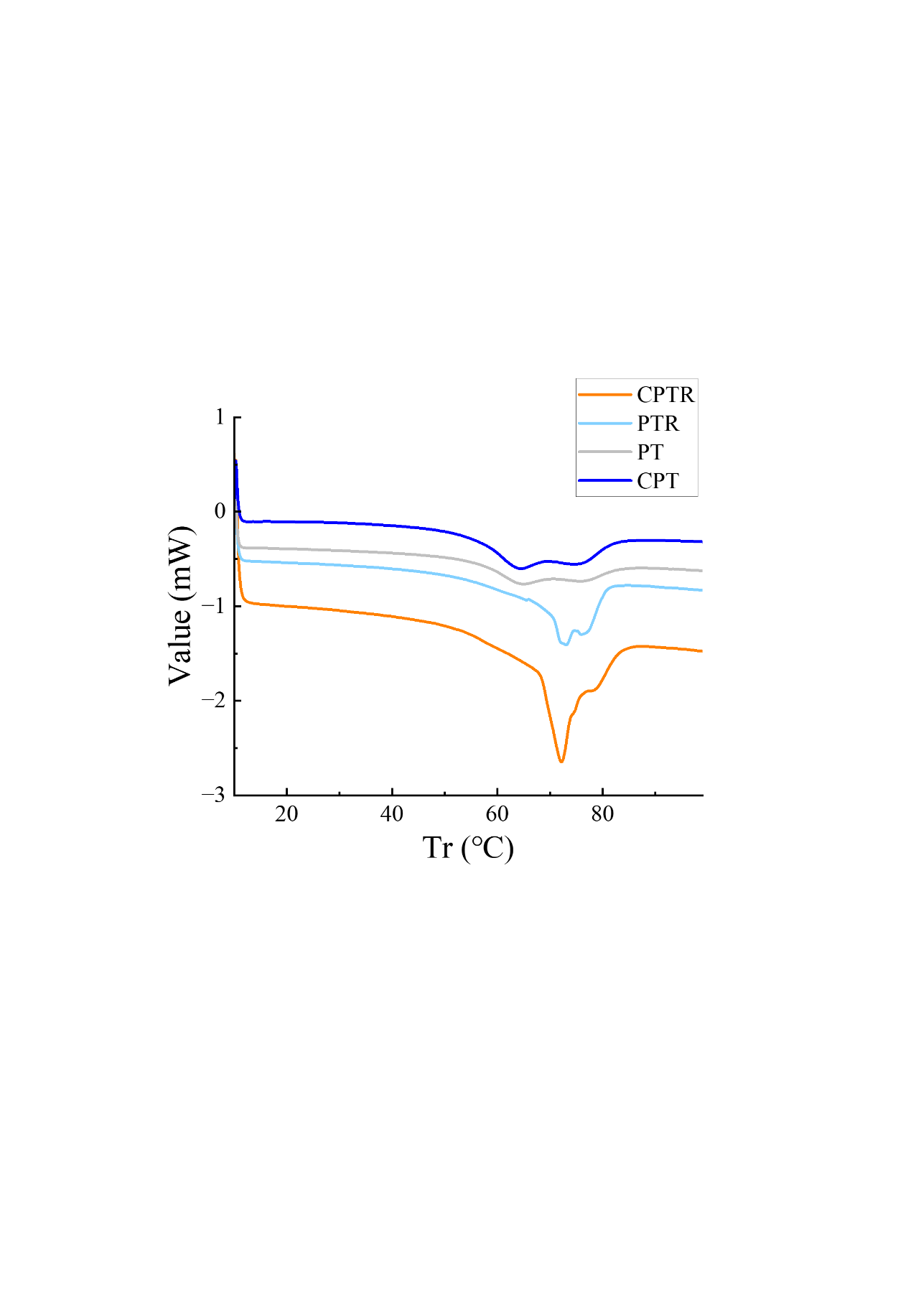
**

**Figure S1.** Original DSC characterization data.

The melting peak results represented the energy absorbed when the ordered structures in the sample disassembled, melted, or denatured upon heating. The more regular the structure, the larger the size, and the more stable the internal hydrogen bonds, the higher the Peak temperature-melting temperature (T_m_) required to disrupt it. Therefore, a higher T_m_ indicated better stability of the ordered structure and stronger performance. Peak area-melting enthalpy (ΔH) represented the total energy required to melt all ordered structures. This value was proportional to the amount of ordered structure in the sample. The larger the ΔH, the more ordered structure there was acting as a reinforcement, and the stronger the performance. Peak depth represented the intensity or cooperativity of the phase transition, with deeper peaks being better


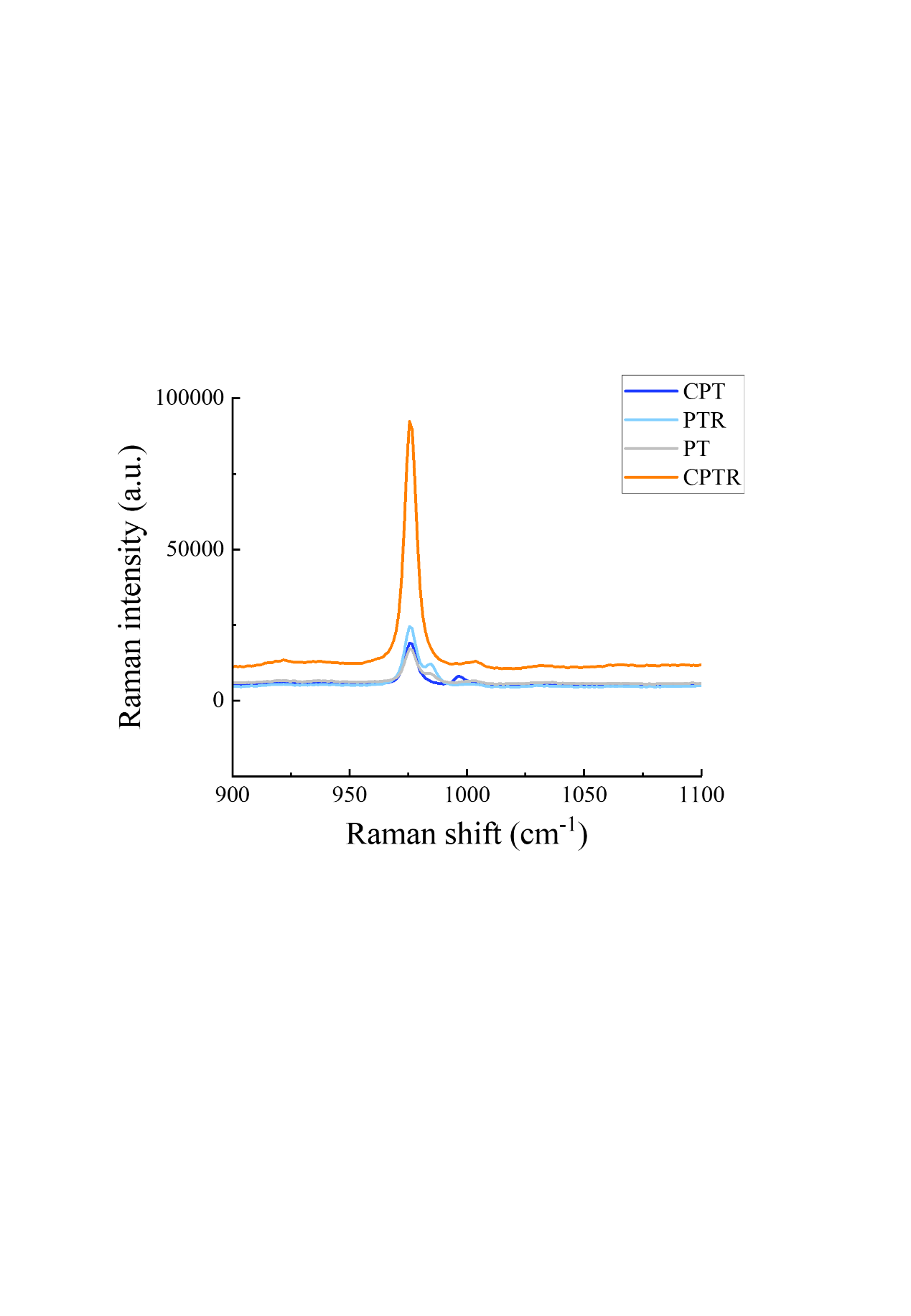


**Figure S2.** Original Raman characterization data.

The sharp peak at a wavelength of 975 cm⁻¹ came from the S-O bond vibration of the sulfate ion. In a hydrogel that had been thoroughly treated, with a denser network and smaller pore size, the internal mass transfer resistance was greater. Ammonium sulfate ions trapped deep within the network were more difficult to wash out through diffusion. The more ammonium sulfate ions remaining in the final sample, the stronger the peak at 975 cm⁻¹, indicating that the original hydrogel network structure was denser and more uniform, with better mechanical properties. Centrifugation made the network more uniform.


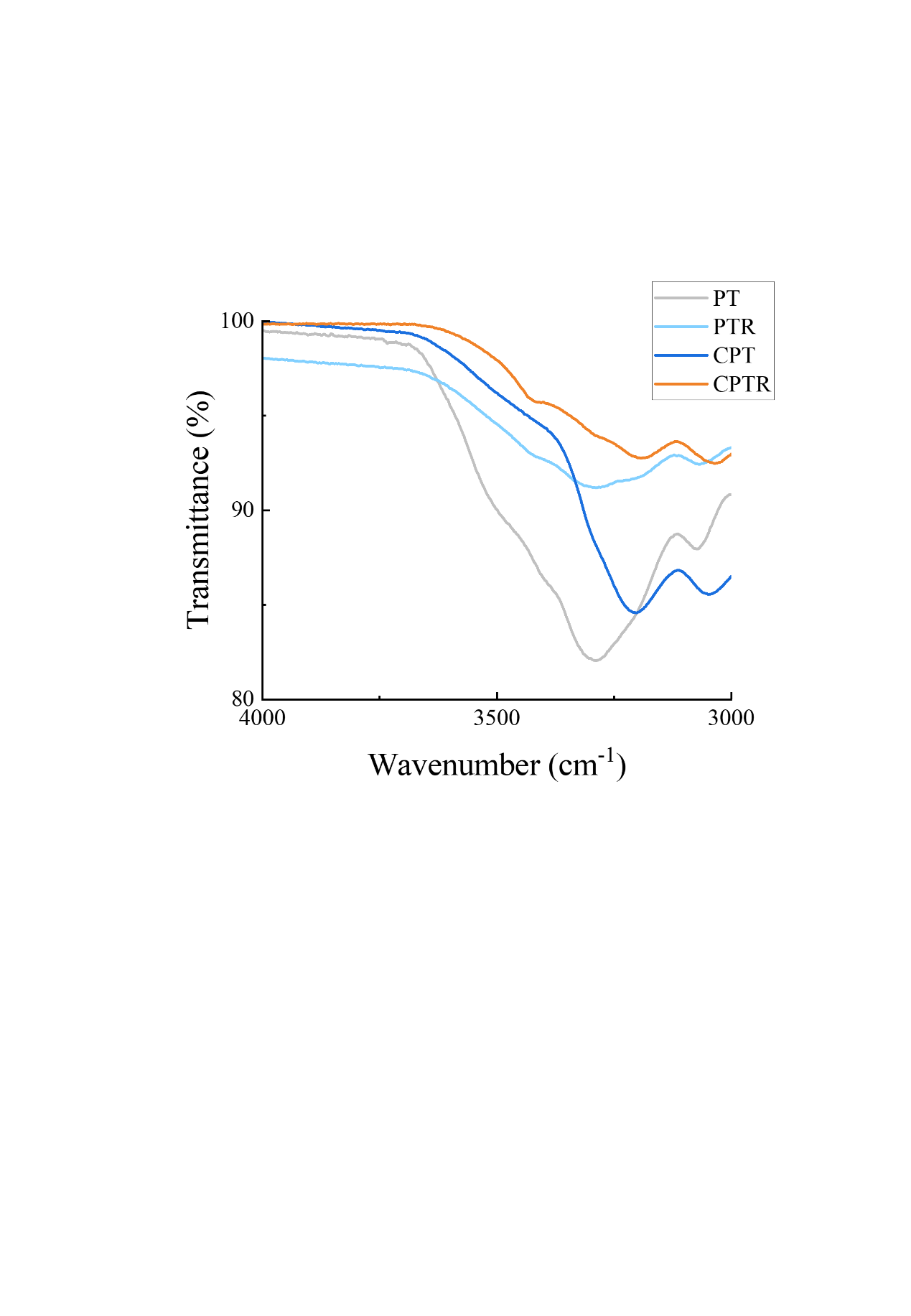


**Figure S3.** Original FTIR characterization data.

In FTIR infrared spectroscopy, the stronger and more numerous the hydrogen bonds were, the more pronounced the red shift and broadening phenomena became, with the red shift being the main factor. The amide A band ( ≈ 3300-3400 cm⁻¹) served as a direct probe of hydrogen bond strength, primarily involving N-H stretching vibrations. When N-H groups participated in hydrogen bonding, their stretching vibration frequency decreased, and the peak became broader and stronger. The stronger the hydrogen bond, the more significant the red shift. Due to the dehydration of ammonium sulfate and the chain aggregation effect, polymer chains were forced closer together, forming a large number of stronger inter chain hydrogen bonds, making the red shift more pronounced. Centrifugation brought the advantage of homogenization, which also promoted the red shift.


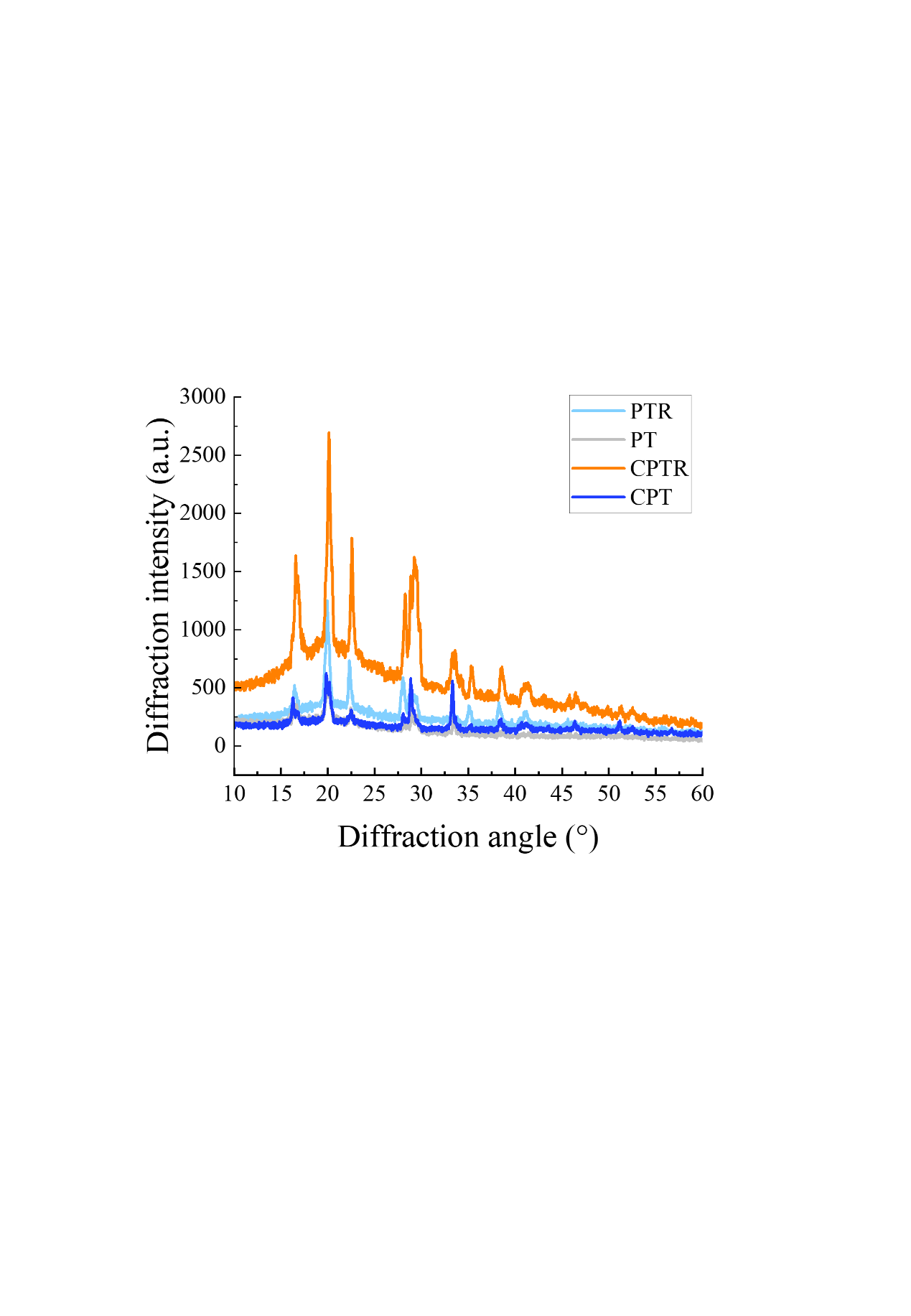


**Figure S4.** Original XRD characterization data.

The changes in the intensity and position of the amorphous halo (Amorphous Halo) at around 20° in the sample reflected changes in density and uniformity of the network. According to the equation of Bragg, this angle corresponded to a distance of approximately d ≈ 0.45 nm. This represented the most common average spacing between molecular chains within the GelMA network. The higher the peak intensity, the more closely packed the molecular chains, the more ordered the network, and the better the network's uniformity/coherence. a shift of the peak to a higher angle indicated better performance. According to the equation of Bragg, the larger the *θ*, the smaller the d (spacing). This meant that the molecular chains were more tightly compressed, the network density was higher, and the network was more compact.


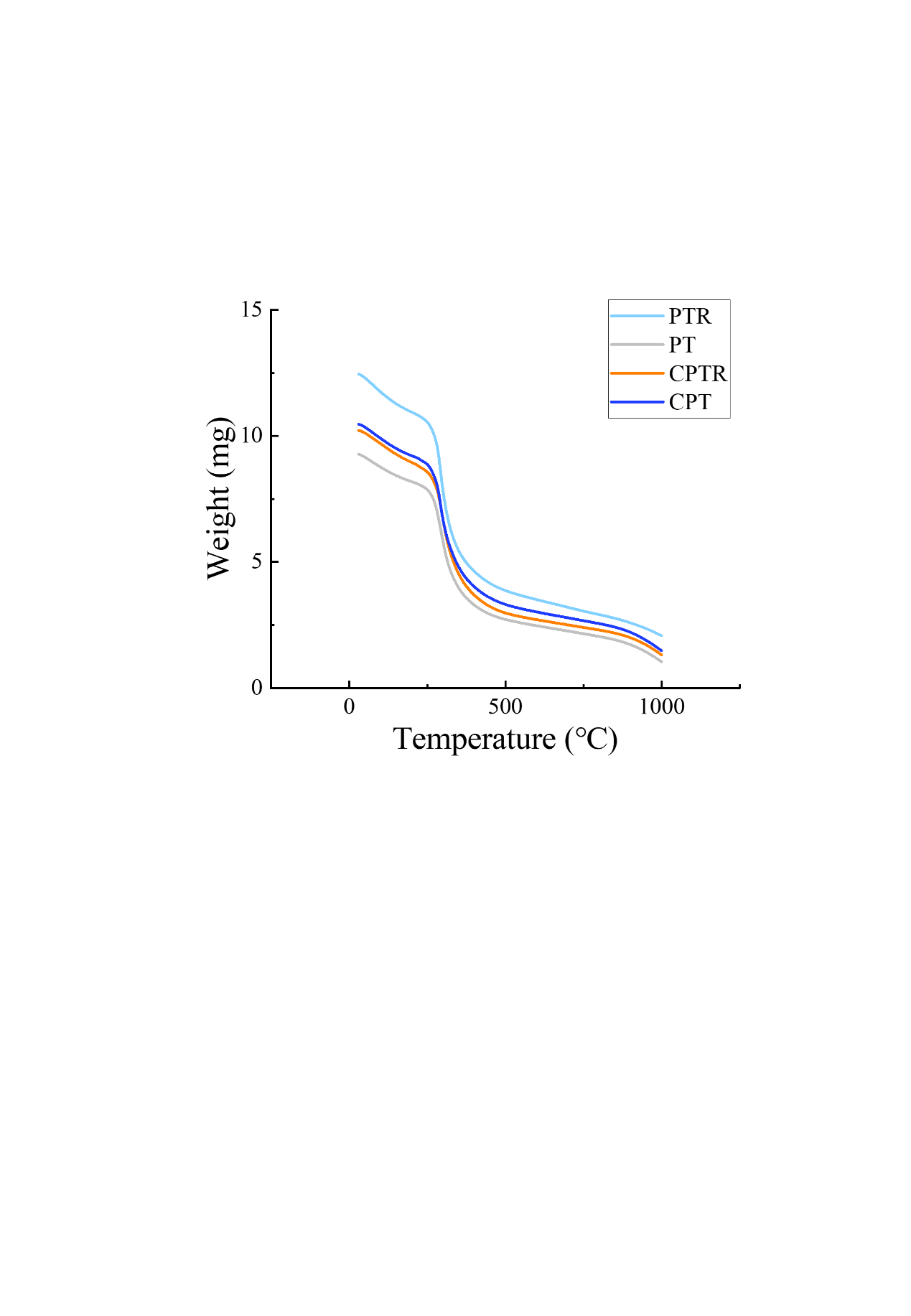


**Figure S5.** Original TGA characterization data.

A sample with better mechanical properties and a denser, more stable network structure had stronger intermolecular interactions, requiring a higher temperature to break these chemical bonds and caused decomposition and volatilization. Therefore, it would exhibit a higher decomposition temperature and less weight loss on the TGA curves. Initial decomposition temperature represented the temperature point at which the material began to undergo irreversible chemical decomposition. It was the most important indicator of thermal stability of a material. A sample with a denser network, stronger intermolecular forced (hydrogen bonding, hydrophobic interactions), and more ordered structures required higher energy (temperature) to initiate the bond breaking process. The higher the initial decomposition temperature, the better the thermal stability of the material, and the stronger its corresponding mechanical properties.


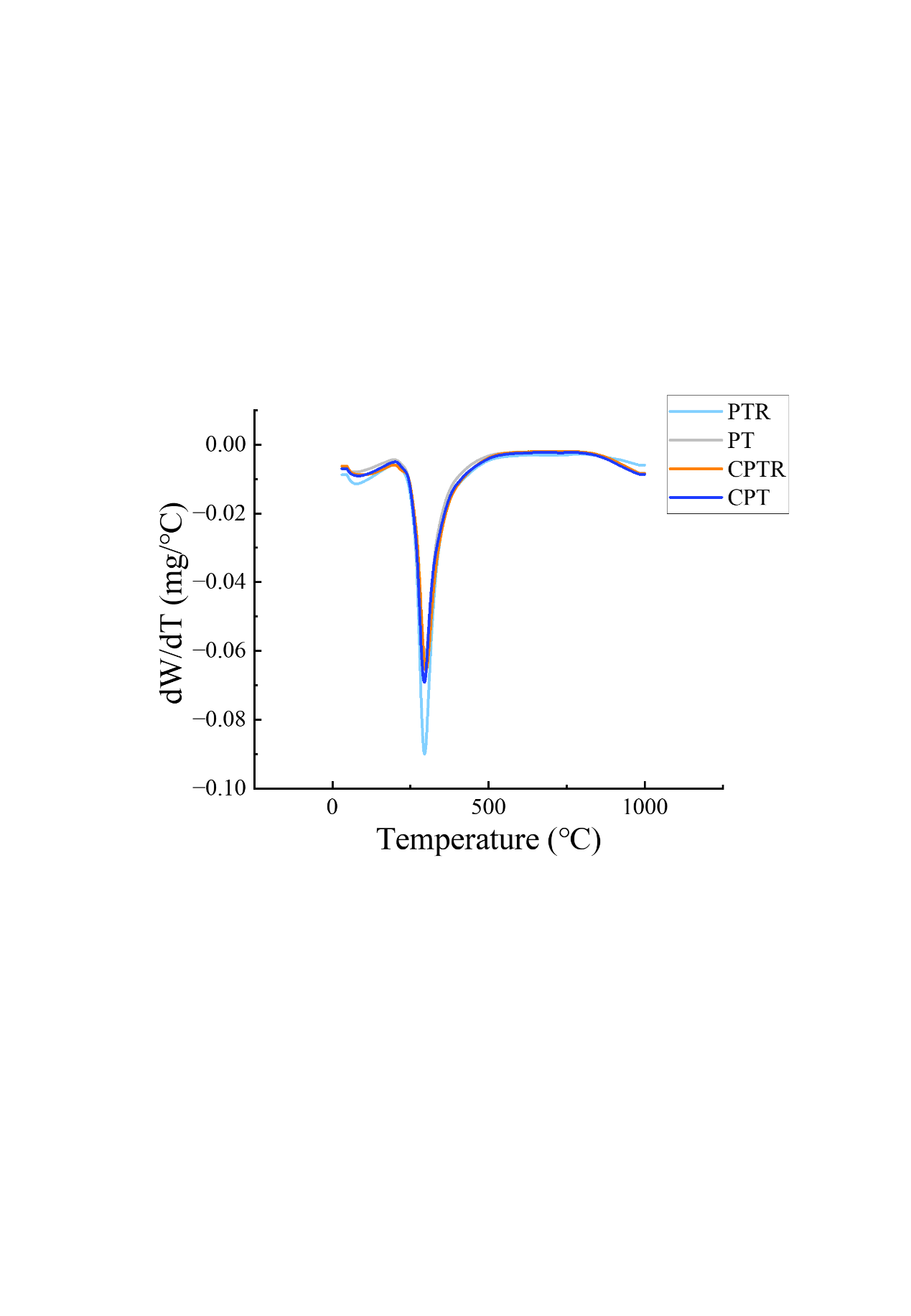


**Figure S6.** Original DTG characterization data.

Temperature at the Maximum Rate of Weight Loss (T_max_) was the temperature corresponding to the peak of the DTG curves, representing the point at which the material decomposes most vigorously. The higher the T_max_, the better the thermal stability of the material.

**
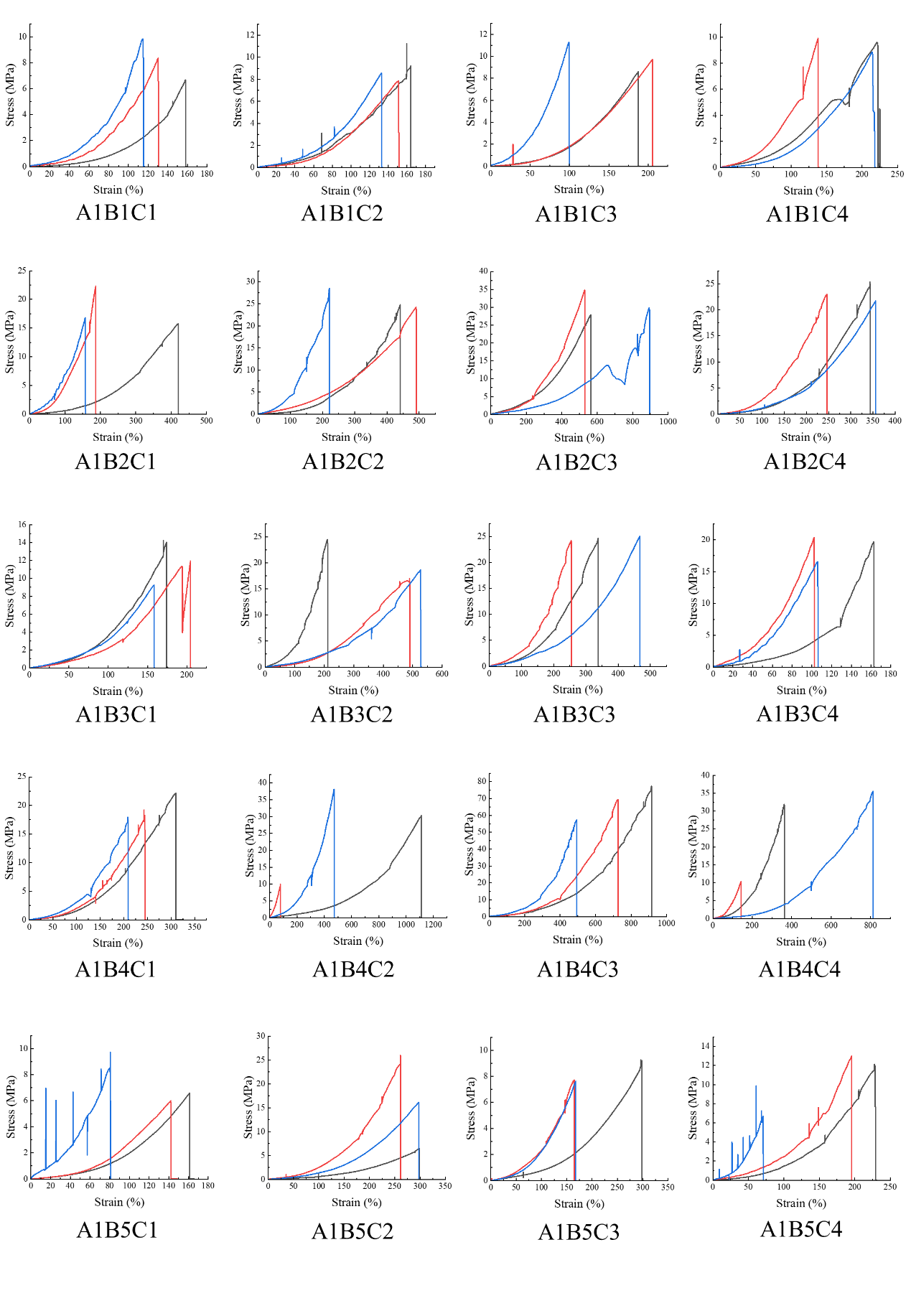
**

**Figure S7.** Original data of mechanical performance tests for different process combinations under non-centrifugal conditions. (A1: no centrifugation. A2: centrifugation. B1: 20 % strain stretching. B2: 30 % strain stretching. B3: 40 % strain stretching. B4: initial 30% strain with an increase of 3 % strain every 5 cycles. B5: initial 30 % strain with an increase of 10 % strain every 5 cycles. C1: no soaking after stretching. C2: soaking for 12 h after stretching. C3: soaking for 24 h after stretching. C4: soaking for 36 h after stretching)

**
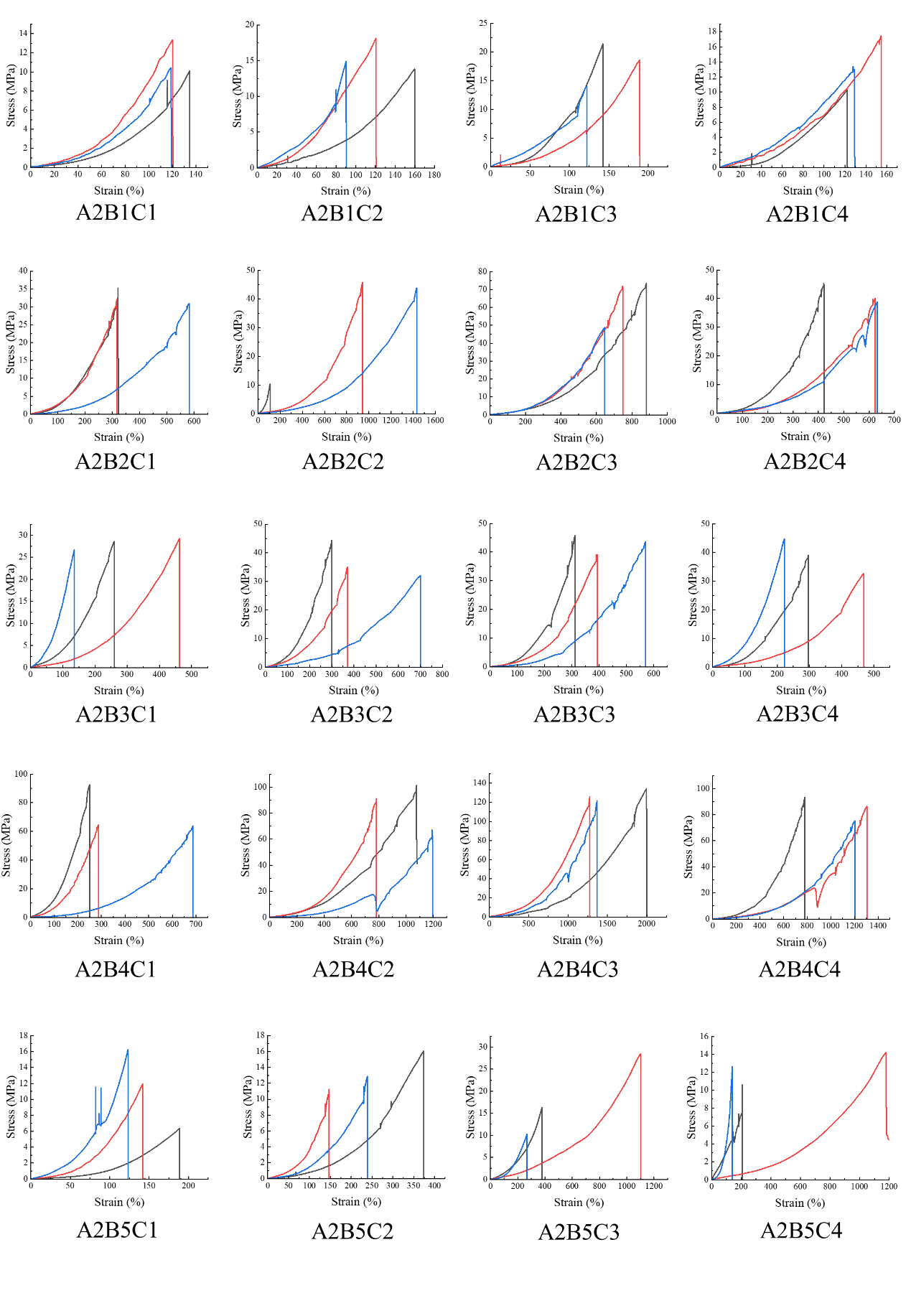
**

**Figure S8.** Original data of mechanical performance tests for different process combinations under centrifugal conditions. (A1: no centrifugation. A2: centrifugation. B1: 20 % strain stretching. B2: 30 % strain stretching. B3: 40 % strain stretching. B4: initial 30% strain with an increase of 3 % strain every 5 cycles. B5: initial 30 % strain with an increase of 10 % strain every 5 cycles. C1: no soaking after stretching. C2: soaking for 12 h after stretching. C3: soaking for 24 h after stretching. C4: soaking for 36 h after stretching)

**Figure S9.** Ashby diagram comparing the mechanical properties of typical tough hydrogels(GP^[1]^, SALT^[2]^, PTC^[3]^, HSH^[4]^, VAHW^[5]^, PU-GBPC^[6]^, SAPAM^[7]^).

The reinforcement strategies of hydrogels are mainly divided into three categories: dynamic sacrificial interactions, intrinsic network structure optimization, and construction of multiscale heterogeneous structures. We selected representative literature from each strategy for comparison. VAWH is a hydrogel reinforced based on the dynamic sacrificial interaction strategy. It has two interpenetrating polymer networks: one provides skeletal support, while the other acts as a sacrificial network that breaks during deformation to efficiently dissipate energy. SAPAM is a hydrogel reinforced based on the intrinsic network structure optimization strategy. By using nano-crosslinkers, network defects are fundamentally eliminated, creating a monodisperse network with uniformly distributed crosslinking points. The harmful dangling chains are converted into effective energy dissipation points through gold-sulfur (Au-S) coordination bonds, thereby optimizing the topology of the monodisperse network. PU-GBPC and HSH are hydrogels reinforced based on a multi-scale heterogeneous structure construction strategy. PU-GBPC is a Janus-structured electronic skin reinforced with PU microfibers, characterized by being ultra-thin, high-strength, highly sensitive, and having good biocompatibility. HSH is formed by inducing nano-clay aggregation in a PVA matrix through a freeze-thaw process, creating a unique phase separation–self-assembly microstructure of polymer-particle mixed aggregates embedded in a continuous matrix, thereby enhancing mechanical performance. PTC and SALT are hydrogels primarily strengthened based on mechanical training and salting-out effects within the multi-scale heterogeneous structure construction strategy. Their strengthening mechanisms have some relevance to the CPTR hydrogel in this study, but they rely on a single strengthening factor and have poor synergistic effects. GP is a hydrogel guided by environmental adaptability design, focusing on optimizing water retention, ionic conductivity, temperature-responsive in situ gelation, and other biointerface-compatible functions; therefore, it is not classified under strengthening strategies and is only used for comparing the mechanical performance of hydrogels guided by environmental adaptability design. The strengthening mechanism of the CPTR hydrogel in this study belongs to the synergistic enhancement of three strengthening strategies (Centrifugal operations are based on dynamic sacrificial interactions, while progressive stretching training and soaking are based on the intrinsic optimization of network structures and the construction of multi-scale heterogeneous structures). Comparisons show that the tensile stress of the CPTR hydrogel in this study is far greater than that of the other hydrogels, with the best overall performance.


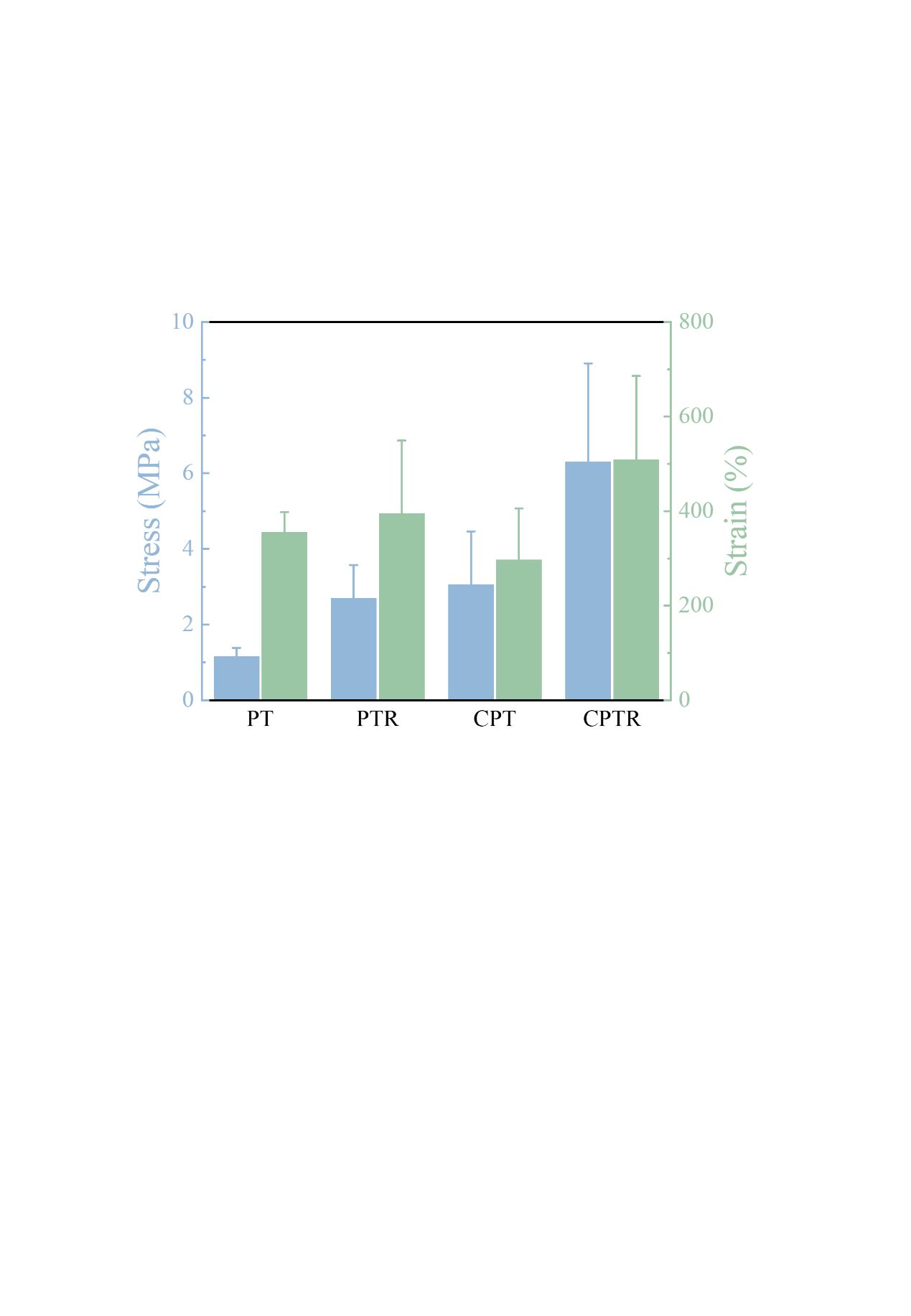


**Figure S10.** Toughness testing under different process combinations.


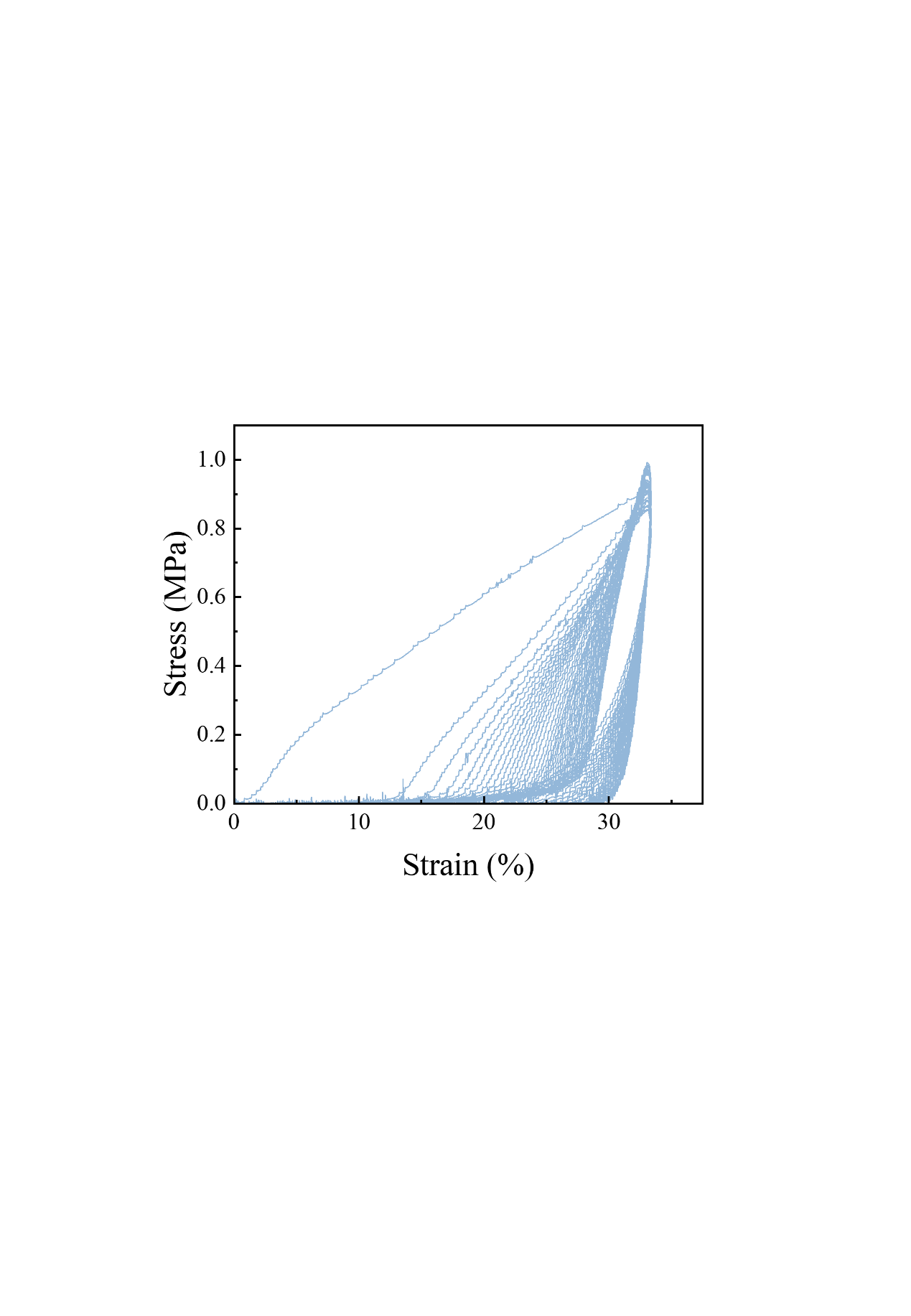


**Figure S11.** Fatigue testing of CPTR biohydrogels.


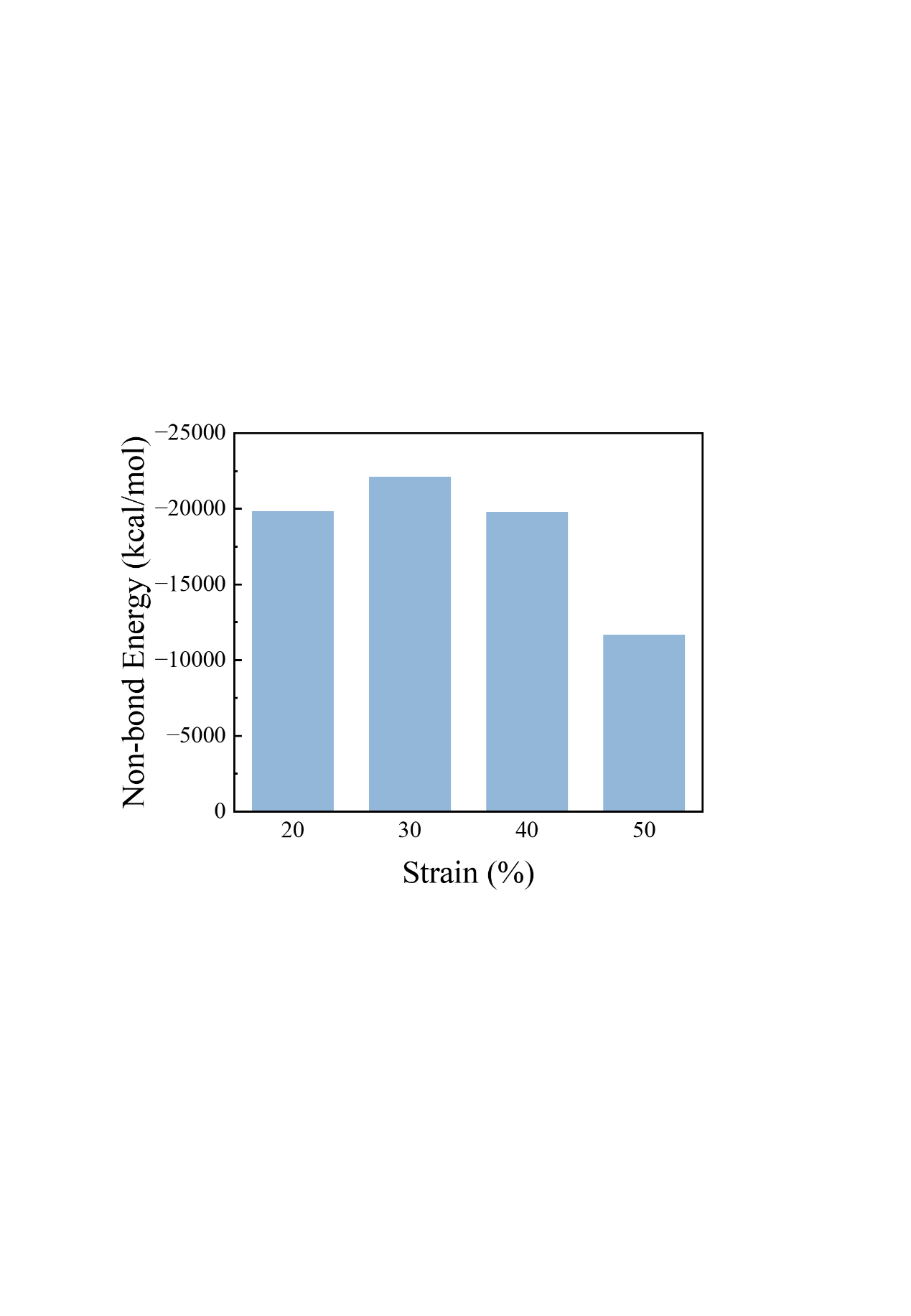


**Figure S12.** Based on MS simulation results of intermolecular interaction forces under different degrees of stretching.

We first used the Forcite module to perform Geometry Optimization on the constructed molecular chains to obtain stable configurations. Then, using Materials Studio software combined with the Perl script StressStrain.pl, we conducted uniaxial stretching simulations on the GelMA molecular chain system under the COMPASSIII force field. By fixing one end of the molecular chain and applying gradually increasing strain to the other end, we considered that after stretching, the molecular chain may be in a random high-energy conformation, and the instantaneous strong repulsive forces between groups due to too close distances would affect their true attractive interactions. This includes the huge internal stress (bond energy) caused by forcibly distorted bond lengths and bond angles. The non-bonded energy also contains unreasonable spatial repulsion, and the strength of intermolecular interactions usually refers to the system’s property at equilibrium or metastable state. Therefore, after each stretching, geometry optimization was performed again to eliminate conformational tension, and the final energy was recorded. The Forcite module of Materials Studio decomposes the total energy of a system into Valence energy and Non-bond energy based on the force field. Valence Energy mainly includes bond stretching, angle bending, dihedral torsion, etc. During single-chain stretching, the changes in this part of the energy mainly come from the changes in chain conformation and slight bond deformation. Non-bond Energy mainly includes van der Waals and Electrostatic interactions. For neutral molecular chains, van der Waals forces are usually dominant. Therefore, the magnitude of non-bond energy directly reflects the strength of intra- and intermolecular interactions (between atoms not connected by covalent bonds). By recording the magnitude of non-bond energy, one can compare the strength of intermolecular interactions. At 20%, 30%, 40%, and 50% strain, the non-bond energy is -19848.668 kcal/mol, -22122.296 kcal/mol, -19797.247 kcal/mol, and -11703.376 kcal/mol, respectively. Therefore, at a 30% tensile strain, the absolute value of non-bond energy is larger, indicating stronger intermolecular interaction


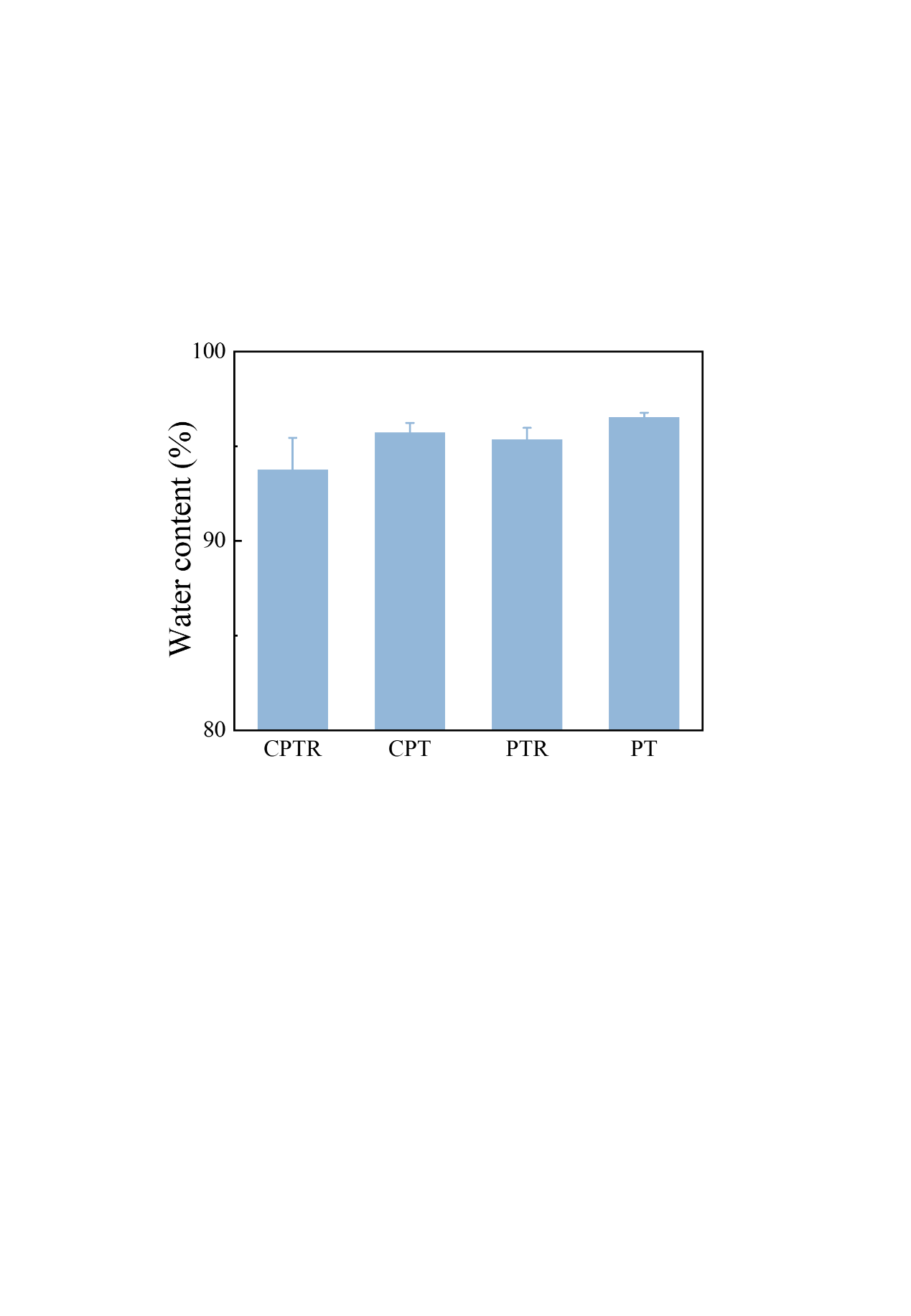


**Figure S13.** Water content under different process combinations.


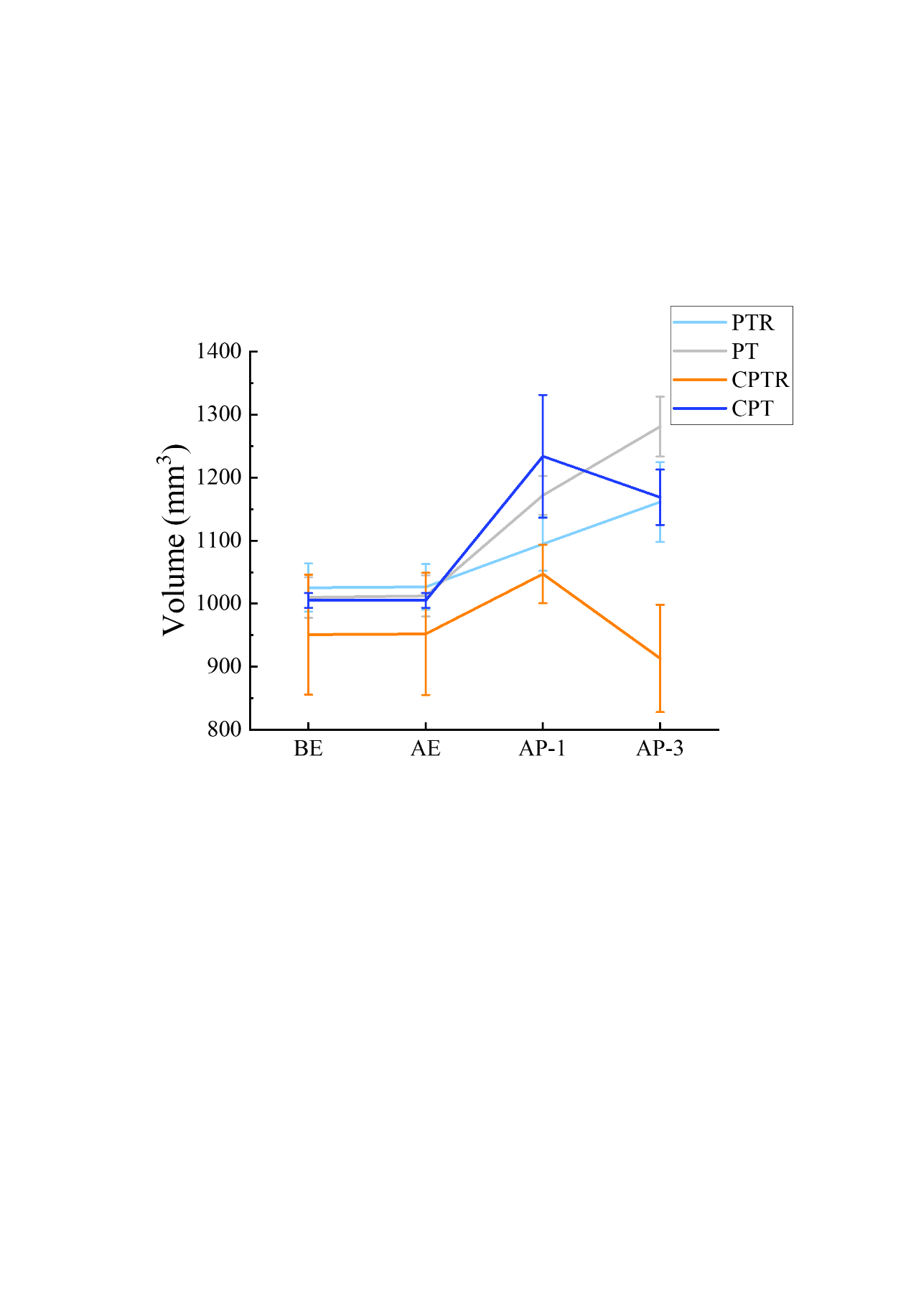


**Figure S14.** Volume changes under different process combinations ( “BE” refers to CPTR biohydrogels before soaking in EGDMA. “AE” refers to CPTR biohydrogels after soaking in EGDMA. “AP-1” refers to CPTR biohydrogels after being soaked in PBS for 1 days. “AP-3” refers to CPTR biohydrogels after being soaked in PBS for 3 days).


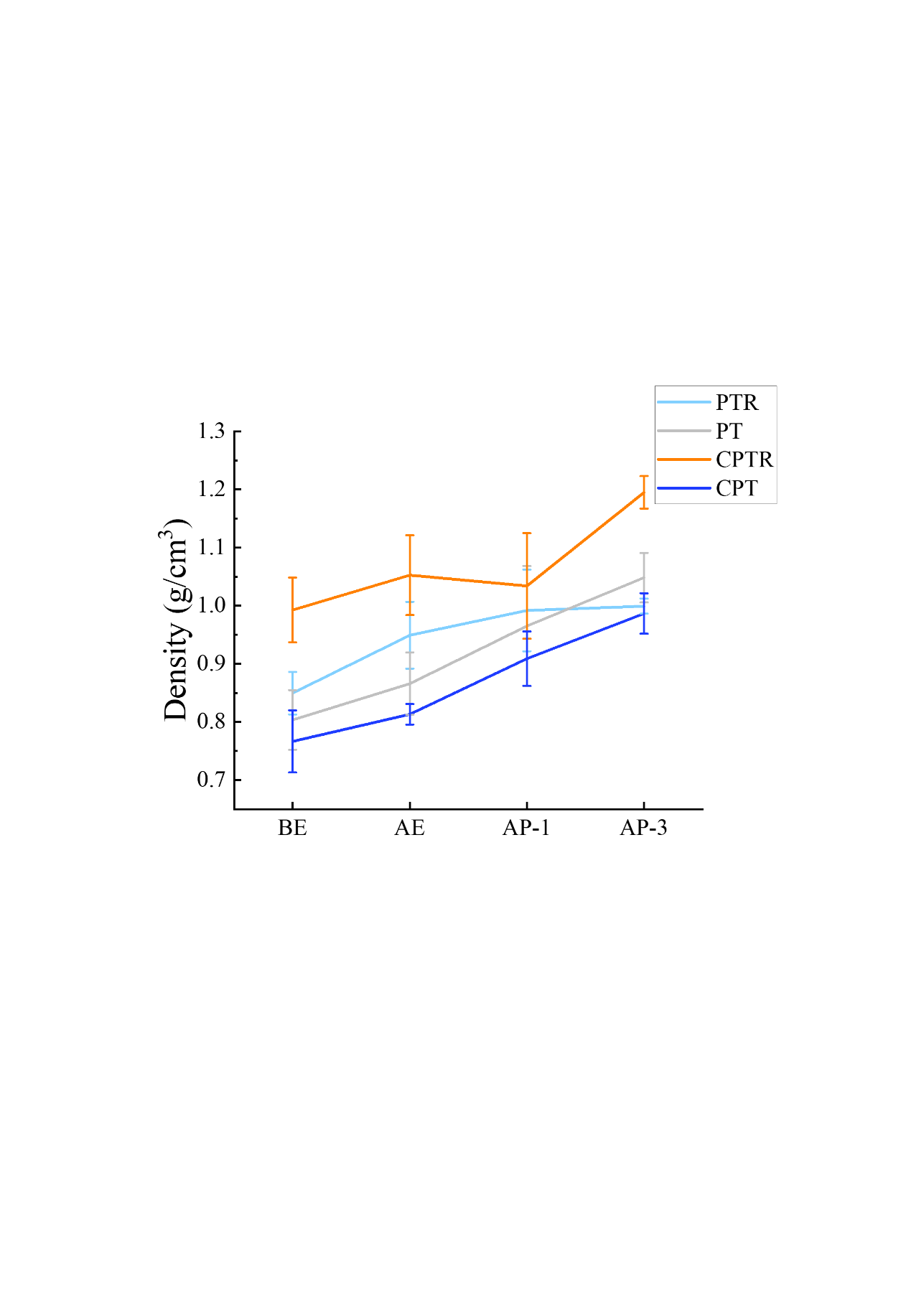


**Figure S15.** Density changes under different process combinations( “BE” refers to CPTR biohydrogels before soaking in EGDMA. “AE” refers to CPTR biohydrogels after soaking in EGDMA. “AP-1” refers to CPTR biohydrogels after being soaked in PBS for 1 days. “AP-3” refers to CPTR biohydrogels after being soaked in PBS for 3 days).


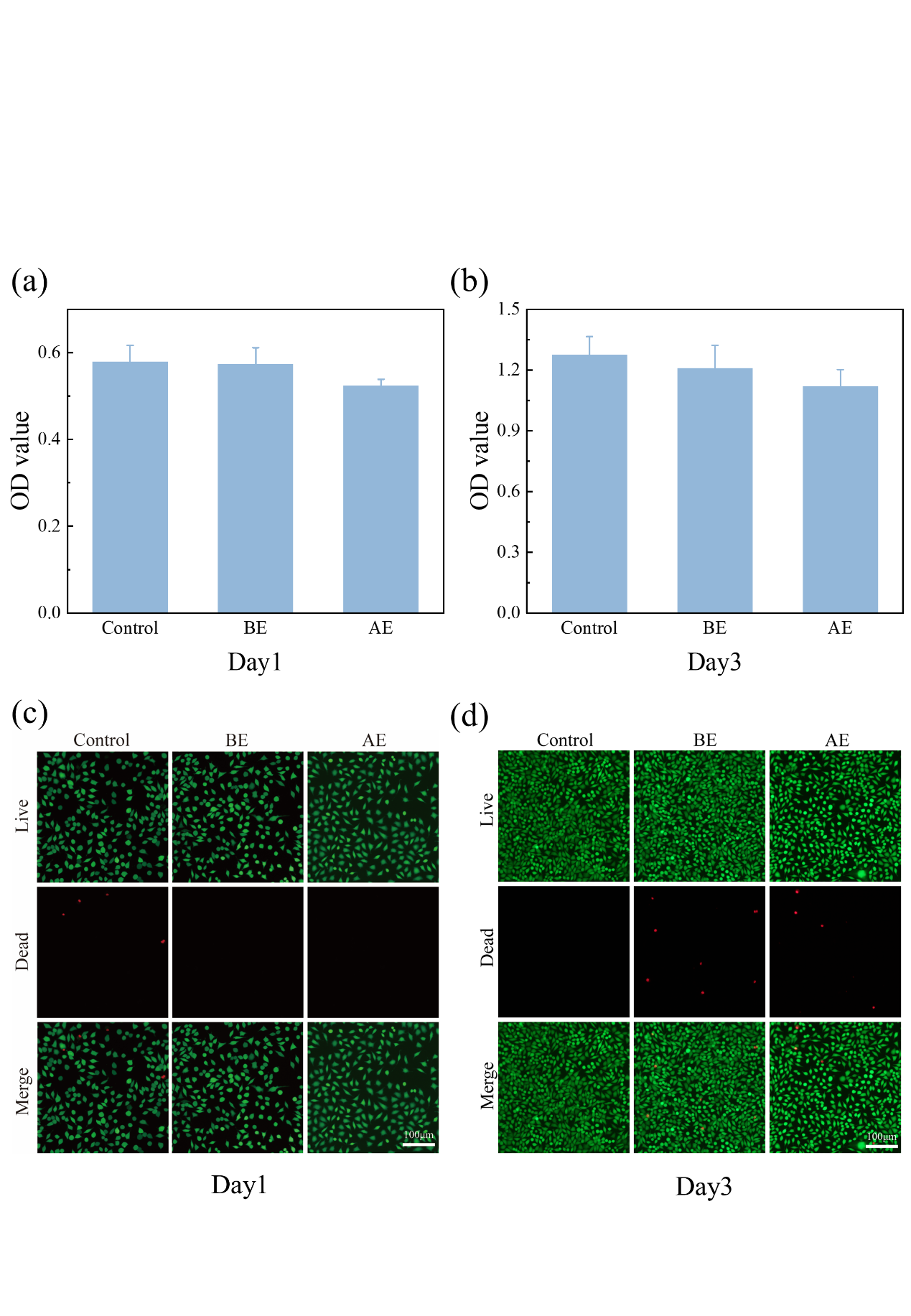


**Figure S16.** Cell viability assay of CPTR biohydrogels. **a, b.** Evaluation of hydrogel cytocompatibility by CCK-8 assay. **c, d.** Cell viability and death staining experiment.( “BE” refers to CPTR biohydrogels before soaking in EGDMA, and “AE” refers to CPTR biohydrogels after soaking in EGDMA)


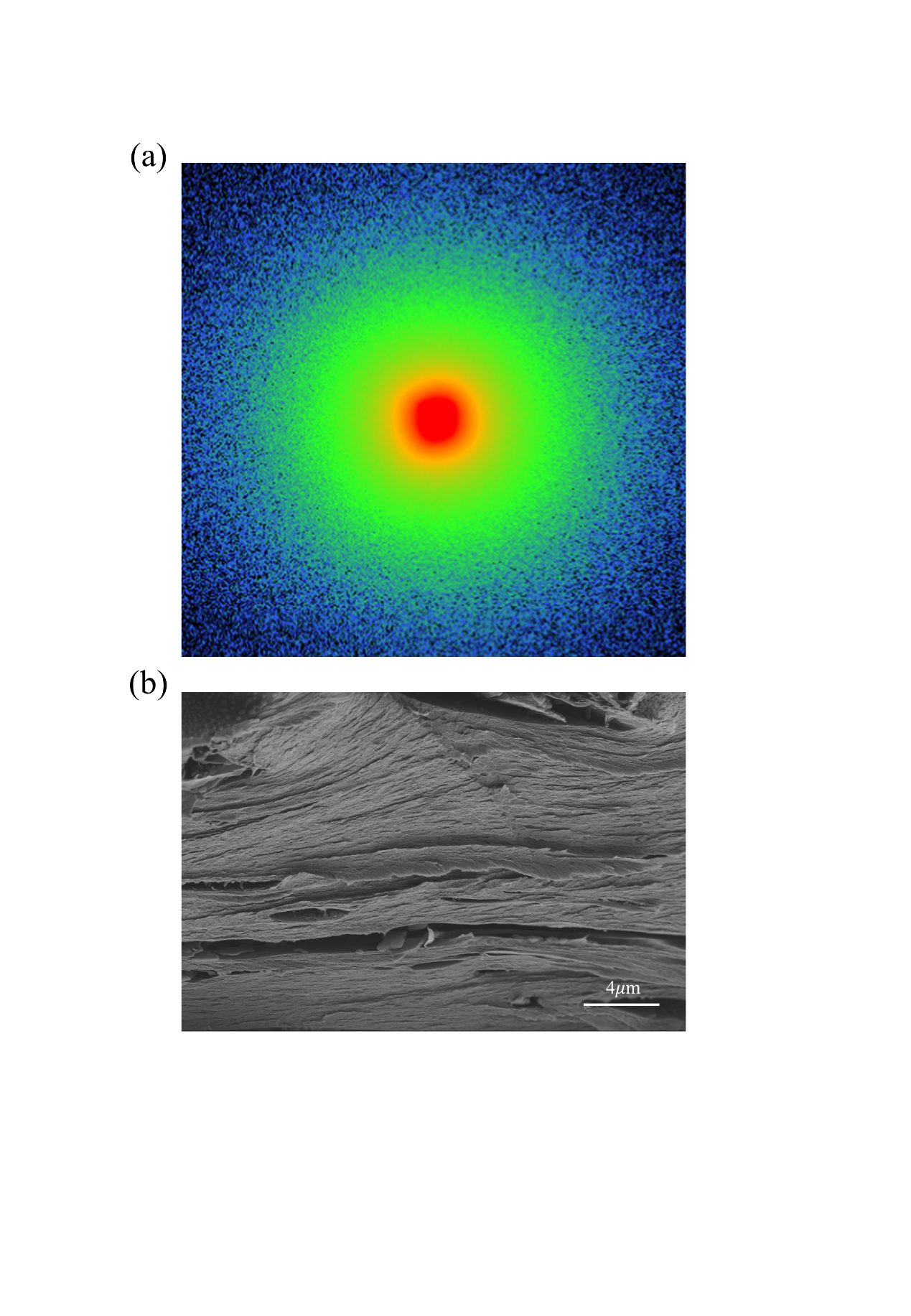


**Figure S17.** SAXS and SEM characterization results of untreated hydrogels. **a.** SAXS characterization results. **b.** SEM characterization results.

**Figure S18.** Comparison of the elastic modulus of CPTR and PTC^[3]^ after normalization by polymer density.

**Figure S19.** Comparison of toughness under different process combinations.

**Figure S20.** Comparison of toughness normalized by polymer density under different process combinations.

**Figure S21.** Comparison of E-modulus normalized by polymer density under optimal solutions of different process combinations.

**Figure S22.** Comparison of E-modulus normalized by polymer density under different process combinations including B1 process.

**Figure S23.** Comparison of E-modulus normalized by polymer density under different process combinations including B2 process.

**Figure S24.** Comparison of E-modulus normalized by polymer density under different process combinations including B3 process.

**Figure S25.** Comparison of E-modulus normalized by polymer density under different process combinations including B4 process.

**Figure S26.** Comparison of E-modulus normalized by polymer density under different process combinations including B5 process.

**References**

1. Lan, Lingyi, et al. Skin‐inspired all‐natural biogel for bioadhesive interface. *Advanced Materials* 36.25 (2024): 2401151.
2. Sun, Xia, et al. A biomimetic “salting out—alignment—locking” tactic to design strong and tough hydrogel. *Advanced Materials* 36.25 (2024): 2400084.
3. Yao, Ke, et al. 3D printing of tough hydrogel scaffolds with functional surface structures for tissue regeneration. *Nano-Micro Letters* 17.1 (2025): 27.
4. Shi, Diwei, Donghwan Ji, and Jinhye Bae. Hierarchically structuralized hydrogels with ligament-like mechanical performance. *Nature communications* (2025).
5. Yin, Chenxiao, et al. High Latent‐Heat, Soft yet Robust Crystalline Salogels via Dual‐Network Design and Solvent Engineering. *Advanced Materials* 37.45 (2025): e10985.
6. Ding, Yarong, et al. A Microfiber‐Reinforced Janus Hydrogel E‐Skin With Recyclable Feature for Multimodal Sensing and Gender‐Specific Physiological Monitoring. *Advanced Science* 13.17 (2026): e20336.
7. Xia, Yuanyuan, et al. Ultra‐Tough Single‐Network Hydrogels via the Synergy of Defect Elimination and Dual Crosslinking. *Advanced Materials* 37.35 (2025): 2417795.
